# CD73 controls ocular adenosine levels and protects retina from light-induced phototoxicity

**DOI:** 10.1007/s00018-022-04187-4

**Published:** 2022-02-25

**Authors:** Karolina Losenkova, Akira Takeda, Symantas Ragauskas, Marc Cerrada-Gimenez, Maria Vähätupa, Simon Kaja, Marius L. Paul, Constanze C. Schmies, Georg Rolshoven, Christa E. Müller, Jouko Sandholm, Sirpa Jalkanen, Giedrius Kalesnykas, Gennady G. Yegutkin

**Affiliations:** 1 MediCity Research Laboratory and InFLAMES Flagship, University of Turku, Tykistökatu 6A, 20520 Turku, Finland; 2 Experimentica UAB, Vilnius, Lithuania; 3 Experimentica Ltd., Kuopio, Finland; 4 Department of Ophthalmology, Loyola University Chicago, Stritch School of Medicine, Maywood, IL USA; 5 Pharma Center Bonn, Pharmaceutical Institute, Pharmaceutical and Medicinal Chemistry, University of Bonn, Bonn, Germany; 6 Turku Bioscience Centre, University of Turku and Åbo Akademi University, Turku, Finland

**Keywords:** NTPDase1/CD39, Ecto-5′-nucleotidase/CD73, Adenosine receptors, Mouse and human retina

## Abstract

ATP and adenosine have emerged as important signaling molecules involved in vascular remodeling, retinal functioning and neurovascular coupling in the mammalian eye. However, little is known about the regulatory mechanisms of purinergic signaling in the eye. Here, we used three-dimensional multiplexed imaging, in situ enzyme histochemistry, flow cytometric analysis, and single cell transcriptomics to characterize the whole pattern of purine metabolism in mouse and human eyes. This study identified ecto-nucleoside triphosphate diphosphohydrolase-1 (NTPDase1/CD39), NTPDase2, and ecto-5′-nucleotidase/CD73 as major ocular ecto-nucleotidases, which are selectively expressed in the photoreceptor layer (CD73), optic nerve head, retinal vasculature and microglia (CD39), as well as in neuronal processes and cornea (CD39, NTPDase2). Specifically, microglial cells can create a spatially arranged network in the retinal parenchyma by extending and retracting their branched CD39^high^/CD73^low^ processes and forming local “purinergic junctions” with CD39^low^/CD73^−^ neuronal cell bodies and CD39^high^/CD73^−^ retinal blood vessels. The relevance of the CD73–adenosine pathway was confirmed by flash electroretinography showing that pharmacological inhibition of adenosine production by injection of highly selective CD73 inhibitor PSB-12489 in the vitreous cavity of dark-adapted mouse eyes rendered the animals hypersensitive to prolonged bright light, manifested as decreased a-wave and b-wave amplitudes. The impaired electrical responses of retinal cells in PSB-12489-treated mice were not accompanied by decrease in total thickness of the retina or death of photoreceptors and retinal ganglion cells. Our study thus defines ocular adenosine metabolism as a complex and spatially integrated network and further characterizes the critical role of CD73 in maintaining the functional activity of retinal cells.

**Supplementary Information:**

The online version contains supplementary material available at 10.1007/s00018-022-04187-4.

## Introduction

Extracellular ATP and its metabolites ADP and adenosine (ADO) are important signaling molecules involved in a wide range of (patho)physiological activities in virtually all organs and tissues [1], including the eye [2–5]. ATP released from damaged neurons, blood vessels, activated microglia, and Müller glial cells triggers diverse proinflammatory, neurodegenerative, and angiogenic processes which are mediated by activation of metabotropic (P2Y) and ligand-gated (P2X) nucleotide receptors expressed in the retina and other ocular structures [2, 3, 6]. Another mechanism of ATP action is conveyed via its ectoenzymatic breakdown into ADO, which in turn binds to adenosine receptors (AR) that function by activating (A_2A_R and A_2B_R) or inhibiting (A_1_R and A_3_R) adenylyl cyclase [1]. A_2A_R and/or A_1_R are especially relevant in terms of ocular physiology by playing a crucial role in pathological retinal angiogenesis [7, 8], neuroinflammation [5, 9], modulation of the circadian clockwork [10], photoreceptor coupling [11], retinal, choroid and optic nerve blood flow [12, 13], and also hyperpolarization of retinal ganglion cells (RGC) and protecting them from apoptosis [6, 14].

Along with significant progress in understanding the function of purinergic receptors, recent studies have begun to uncover the complexity of regulatory mechanisms governing the duration and magnitude of purinergic signaling in the eye. Previous research has focused on the expression of key nucleotide-inactivating/ADO-producing enzymes: ecto-nucleoside triphosphate diphosphohydrolase-1 (NTPDase1, also known as CD39), NTPDase2 and ecto-5′-nucleotidase/CD73 in primate [15–17], rodent [3, 8, 17–19], and zebrafish [19, 20] retinas in terms of their role in control of angiogenesis, diabetic retinopathy, intraocular pressure, and neurovascular coupling. In addition, CD73 has been widely employed as a cell surface marker for the enrichment of pluripotent stem cell-derived photoreceptor populations and the isolation of photoreceptors from retinal organoids [21, 22]. Soluble forms of CD73, adenosine deaminase (ADA), adenylate kinase-1 and other enzymes were also identified in the human vitreous fluid, where they coordinately regulate ocular ATP and ADO levels via two counteracting, purine-inactivating and ATP-regenerating, pathways [16, 23]. Multiple human disorders have been linked to abnormalities in purine metabolism, including cancer [24, 25], cardiovascular diseases [26], and ocular diseases [9, 16]. Several potent small-molecule inhibitors and antibodies directed against CD39 and CD73 were developed recently and tested in clinical trials as potential anti-cancer drugs [25, 27, 28]. However, one of the obstacles preventing translation of purinergic enzymes to the clinic is the lack of consideration of redundant pathways controlling ATP and ADO levels in a certain synergistic, counteracting or compensatory manner [29, 30].

The complexity of the architecture and function of the mammalian eye require the development of advanced tools to study the extracellular space in heterogeneous retinal environment. Conventional histological analyses of protein expression performed using formalin-fixed paraffin-embedded tissue sections or cryo-embedded sections can offer high-resolution images, but the limited thickness of slices hampers the acquisition of more information on the *z*-axis. Recent development of advanced platforms such as clearing-enhanced three-dimensional (3D) and other volumetric imaging techniques permits cell-level analysis of cell positioning in the context of macroscale tissue structure [31, 32]. By using a high-resolution 3D multiplexed imaging, in situ enzyme histochemistry and flow cytometric analysis of mouse retina, in combination with single cell transcriptomic data of mouse and human retinal cells, this study was undertaken to assess the whole pattern of purine metabolism in the mammalian eye. Furthermore, we tested pharmacological intervention aimed at reducing intraocular ADO levels by using a novel highly potent and metabolically stable CD73 inhibitor PSB-12489 [27], and demonstrated the essential role of CD73 in protecting the mouse retina from light-induced phototoxicity.

## Materials and methods

### Reagents and antibodies

Detailed information on primary antibodies and other molecular markers used in this study is provided in Table S1. Secondary Alexa Fluor^®^ 488-, 546-, 633- and 750-conjugated goat anti-mouse, anti-rabbit, anti-rat, anti-guinea pig, and anti-chicken antibodies, Alexa Fluor^®^ 546-phalloidin, Alexa Fluor^®^ 647-streptavidin, “Click-iT™ Plus TUNEL Assay for In Situ Apoptosis Detection, Alexa Fluor™ 488 dye”, ProLong^®^ Gold Antifade reagent with 4,6-diamidino-2-phenylindole (DAPI), 5′-bromo-4-chloro-3-indolyl phosphate (BCIP), and nitro blue tetrazolium (NBT) were from Invitrogen™ (ThermoFisher Life Technologies). Cy3™-conjugated donkey anti-guinea pig IgG and Fab fragment donkey anti-mouse IgG were from Jackson Immuno Research Laboratories (West Grove, PA, USA). Brilliant Violet 421™ (BV421)-conjugated streptavidin was from BioLegend^®^ (San Diego, CA, USA). Purified rat anti-mouse CD16/CD32 (Mouse BD Fc Block™) was from BD Pharmingen™. [2,8-^3^H]AMP was from American Radiolabeled Chemicals Inc. (Campro Scientific, The Netherlands). Medetomidine and atipamezole hydrocloride were from CP-Pharma-Handelsgesellschaft mbH (Germany). Tropicamide (Oftan^®^ Tropicamid) and phenylephrine hydrochloride (Oftan^®^ Metaoksedrin) were from Santen (Finland). Ketaminol was from Intervet International B.V (The Netherlands). Low melting temperature agarose (LMA, NuSieve™ GTG™ Agarose) was from Lonza. Biotin-conjugated lectin from *Bandeiraea simplicifolia* (isolectin subunit B4, IB4), collagenase P from *Clostridium histolyticum*, α,β-methylene-ADP (AMPCP), ATP, AMP, and other chemicals were from Sigma-Aldrich.

### Animals

Female and male C57BL/6N mice were obtained from Janvier Labs (France). The animals were maintained at Central Animal Laboratory of the University of Turku (Turku, Finland) and used for histochemical analysis of the eye, flow cytometry and blood serum preparation. The experimental procedures were reviewed by the local Ethics Committee on Animal Experimentation of the University of Turku and approved by the Provincial State Office of Western Finland with the license ID ESAVI/5762/04.10.07/2017. For intravitreal treatment and BL exposure, male C57BL/6JrJ and BALB/c mice (obtained from Janvier Labs, France, and the Laboratory Animal Centre, University of Tartu, Tartu, Estonia, respectively) were maintained at Experimentica Ltd. Laboratory Animal Center (Kuopio, Finland). The animals were treated in accordance with the ARVO Statement for the Use of Animals in Ophthalmic and Vision Research and the EC Directive 86/609/EEC for animal experiments, using protocols approved and monitored by the Animal Experiment Board of Finland (Experimentica Ltd. license ID: ESAVI-004139-2017/ESAVI-10815-2020). All mice were 3–4 months of age, with a body weight of 22–25 g. The animals were raised in pathogen-free conditions, housed at a constant temperature (22 ± 1 °C) in a light-controlled environment (lights on from 7 am to 7 pm), and provided with food and water ad libitum.

### Intravitreal treatment and bright light exposure

C57BL/6JrJ and BALB/c mice (4 animals per group) were used in the first study. Aliquots of PSB-12489 (2 μL of 2 mM) and equal volumes of PBS were administered by intravitreal injections into the right (OD) and left (OS) eyes, respectively. Mice were kept in the dark for 12 h before fERG recording, except for the acute action studies of CD73 inhibitor, where treated mice were dark adapted for only 6 h. In the second study, a total of 24 BALB/c mice were divided into six groups (3–5 animals per group). The animals were kept in transparent plastic cages and subjected to different experimental settings, as outlined below. White light lamps (URZ3372, 6400K, Kemot, Poland) were directed to the cages (one from the bottom and one from the top). Mice were exposed to 9500 lx bright light (BL) for 14 h, starting from 19.00 on day 0 until 9.00 on day 1. Five hours before BL induction, 2 μL aliquots of PSB-12489 (2 mM) were administered by bilateral intravitreal injections to both eyes. Equal volumes of PBS were administered to the eyes of vehicle-treated mice. The animals were returned to a normal facility light/dark cycle of 12 h/12 h and used in experiments 7 days after the treatments, as described below.

### Flash electroretinography (fERG)

Mice were dark adapted for 12 h unless otherwise specified. All procedures for fERG recordings were performed under dim red light. The animals were anesthetized with a mixture of ketaminol (37.5 mg/kg) and medetomidine (0.45 mg/kg) administered intraperitoneally. Body temperature was maintained through the use of a heating pad (set to 38 °C). The pupils were dilated by administering tropicamide, and 3 min later phenylephrine hydrochloride (100 mg/mL). To improve conductivity between eye and electrode, one drop of 0.9% saline was applied to both eyes. The reference electrode needle was inserted in between the eyes and the common grounding electrode was inserted into the base of the tail. Signals were recorded using the Celeris-Diagnosys system (Diagnosys LLC, Massachusetts, USA). To evaluate the functional response from rods and cones, a flash of various intensities (0.003–10 cd s/m^2^) was used (Table S2). General anesthesia via medetomidine was immediately reversed by an α2-antagonist atipamezole (0.5 mg/kg sc.) The following parameters were analyzed and reported: amplitude and latency of the a-waves (first negative fERG component) and amplitude and latency of the b-waves (first positive fERG component). All parameters are provided as raw data.

### Optical coherence tomography (OCT)

High-resolution spectral domain OCT was performed on the baseline and 7 days after exposure of the BALB/c mice to PSB-12489 and/or BL, as described elsewhere [33]. All measurements were performed under general anesthesia. The pupils of both eyes were dilated after application of 5 mg/mL solution of tropicamide. To prevent corneal drying, a Systane Ultra Eye gel (Systane^®^ Ultra, Norbrook, England) was applied on the cornea. The mice were fixed in the holder and ten series of 100 b-measurements were carried out (each b-measurement had 1000 a-measurements). The data obtained were aligned, averaged and a 3D image was created. Photoreceptor layer thickness was measured at 25 different points, which were selected using InVivoVueDiver (Bioptigen, JAV) software. The central point was targeted at the center of the optic nerve. The photoreceptor layer thickness was estimated by measuring the distance between the outer plexiform layer and the external limiting membrane.

### Sample collection and processing

Mice were killed by carbon dioxide and the eyeballs were immediately enucleated and processed for further analyses in the following ways. For enzyme histochemistry, the eyeballs were embedded in the cryo-mold with Tissue-Tek^®^ optimal cutting temperature compound (Sakura Finetek Europe B.V., the Netherlands), cut at 10 μm onto Superfrost^®^ Plus slides (ThermoFischer Life Technologies) using a Leica CM 3050S cryostat, air-dried and stored at − 80 °C. For immunofluorescence staining, the eyes were fixed for 2 h at room temperature (RT) with PBS containing 4% paraformaldehyde (PFA) and embedded in the mold with 4% solution of LMA dissolved in PBS and pre-heated in the microwave oven. LMA-embedded eyes were sectioned at 100 μm thickness using a Leica VT1200S vibrating microtome, additionally fixed for 30 min with 4% PFA, stored in PBS at 4 °C, and processed for 3D immunofluorescence staining within 1 week of preparation. For flow cytometry analysis, the retinas were dissected from the eyecups and digested into the single cell suspension, as described below.

### In situ enzyme histochemistry

For localization of ecto-nucleotidase and TNAP activities, the combined histochemical approach was employed [34]. In brief, tissue cryosections were thawed, fixed for 5 min with 4% PFA, and pre-incubated for 45 min at RT in Trizma-maleate sucrose buffer (TMSB) [40 mmol/L Trizma-maleate, 0.25 mol/L sucrose, pH 7.4] supplemented with the TNAP inhibitor tetramisole (2 mM). The slides were subsequently incubated for 1 h at RT in a mixture containing TMSB (pH 7.4), 2 mM tetramisole, 2 mM Pb(NO_3_)_2_, 0.5 mM CaCl_2_ and one of the following nucleotide substrates: ATP (300 µmol/L), ADP (300 µmol/L) and AMP (1 mmol/L). In blank specimens, the substrate was omitted from the incubation solution. The lead orthophosphate precipitated in the course of nucleotidase activity was visualized as a brown deposit by incubating the sections for 15 s in 0.5% (NH_4_)_2_S. TNAP activity was additionally evaluated by measuring the intensity of dark purple precipitate after incubating the tissues for 20 min at RT in a mixture containing TMSB (pH 9.3), 5 mM MgSO_4_ and artificial enzyme substrates BCIP and NBT (2 mmol/L each). Tissue sections were also stained with hematoxylin and eosin (H&E). Whole tissue section imaging was performed using Pannoramic-250 Flash slide scanner (3DHistech Ltd., Budapest, Hungary) with a 20 × objective.

### Immunofluorescence staining

LMA-embedded vibratome-cut eye sections (100–150 µm thickness) were incubated for 1 h at RT in 300 μL PBS containing 2% bovine serum albumin (BSA) and 0.5% (vol/vol) Triton X-100 (blocking buffer) and subsequently incubated overnight at 4 °C with biotin-conjugated IB4 and primary antibodies diluted in 300 μL of blocking buffer, as specified in Table S1. To avoid off-target background signal during staining of mouse eyes with mouse anti-rhodopsin and anti-NeuN antibodies, endogenous immunoglobulins were blocked by pretreating the samples for 2 h with unconjugated Fab fragment of donkey anti-mouse IgG (20 μg/mL). Negative control staining was also performed in which eye sections were incubated with isotype-matched pre-immune sera from rabbit and guinea pig used at the same dilutions as the primary anti-CD73 and anti-CD39 antibodies. The samples were incubated overnight at 4 °C with the appropriate fluorochrome-conjugated secondary antibodies and Fluor^®^ 647-streptavidin diluted in blocking buffer at ~ 1:800. Alexa Fluor^®^ 488-conjugated anti-CD31, NL493-conjugated anti-tubulin-βIII, and Cy3™-conjugated anti-smooth muscle actin-α (SMA-α) antibodies and Alexa Fluor^®^ 546-Phalloidin were added during the incubation with secondary antibodies for labeling the vascular endothelial cells, neuronal filaments, perivascular cells, and F-actin filaments, respectively. All staining procedures were performed in a 24-well plate under 60 rpm orbital rotation, by washing the wells after each treatment with 300 μL of blocking buffer (3 × 30 min). Stained eye sections were additionally washed for 10 min in 500 μL PBS, transferred onto the microscope slide, aligned using forceps under stereomicroscope, and mounted with ProLong^®^ medium with glass spacers inserted between the slide and the coverslip. Imaging was performed using 3i CSU-W1 spinning disk confocal microscope (Intelligent Imaging Innovations, Inc.) equipped with Hamamatsu ORCA Flash 4 sCMOS camera (Hamamatsu Photonics, Hamamatsu, Japan) and Slidebook 6.0 software. Z-stacks of the medial retina and other eye structures were captured using the following objectives: Plan-Apochromat 10 ×/0.45, Plan-Apochromat 20 ×/0.8, LD C-Apochromat 40 ×/1.1, and Plan-Neofluar oil 63 ×/1.4. Maximum intensity projections and 3D reconstructed images were prepared using Imaris 8.4 software (Bitplane). 3D datasets were rendered into movies using Imaris Animation technology and exported to mp4 format.

To study the effect of CD73 inhibitor and BL exposure on photoreceptor CD73 expression, eye tissue cryosections were incubated for 2 h at RT with anti-CD73 and anti-rhodopsin antibodies (diluted at 1:300 and 1:1000, respectively), and subsequently incubated for one hour with the appropriate fluorochrome-conjugated secondary antibodies. Stained eye sections were mounted with ProLong^®^ medium with DAPI and imaged using Pannoramic Midi Fluoresence slide scanner (3DHistech Ltd., Budapest, Hungary).

### Flow cytometry

Eyes were gently enucleated from C57BL/6N mice. The cornea and lens were removed and retina was carefully dissected from the eyecup using a dissection microscope, fine forceps and surgical microscissors. Retinas from two mice were pooled and digested for 1 h at 37 °C with RPMI-1640 medium containing 0.2 mg/mL collagenase P and 0.1 mg/mL DNase. The cells were passed through a 100 µm filter and blocked for 15 min with purified rat anti-mouse CD16/CD32 (Mouse BD Fc Block™). The single cell suspension was subsequently incubated for 20 min with anti-CD73 and anti-CD39 antibodies (or isotype-matched immunoglobulins), together with biotin-conjugated IB4 and fluorescently-labeled antibodies against CD45, CD11b, P2Y_12_R, and CD31 (see Table S1). After washing, secondary antibodies including Cy3™- or Alexa Fluor^®^ 633-conjugated donkey anti-guinea pig IgG, Alexa Fluor^®^ 488-conjugated goat anti-rabbit IgG, and a BV421™-conjugated streptavidin were added. Stained cells were washed and fixed with 2% paraformaldehyde for 10 min. Flow cytometry analyses were performed using BD LSRFortessa (BD Biosciences) and analyzed using FlowJo software (TreeStar Inc).

### Competitive CD73 assays and analysis of photoreceptor AMPase activity

Soluble CD73 activity was determined by thin layer chromatography (TLC) using human and mouse sera as enzyme sources and [^3^H]AMP as preferred enzyme substrate [27]. The effect of CD73 inhibitors on photoreceptor AMPase activity was also evaluated in situ by using lead nitrate-based enzyme histochemistry [34, 35]. Noteworthy, both AMPCP and PSB-12489 act as reversible CD73 inhibitors. Therefore, it was necessary to maintain these compounds in the assay medium during both the pre-treatment step (30 min at room temperature) and subsequent 1-h incubation of the mouse eye cryosections with the AMP substrate. AMPase activity was determined by measuring AMP-specific brown staining intensities from the whole slide images using QuPath v.0.2.3 software [36]. Shortly, a project including all images was created. Tissue areas were detected using the threshold classifier. The classifier was run with full resolution (240 nm/pixel) for the whole project. The DAB (3,3′-diaminobenzidine) channel and Gaussian pre-filtering were selected. The threshold was set to 1.15 without smoothing and areas above the threshold were classified as “positive”. Representative areas of the OPL, ONL and OS of the photoreceptor layer were manually selected, and the average DAB intensity level was used for AMPase intensity quantification. The script for the analysis is shown in Table S3*.*

### Quantification of cell nuclei in the retinal layers

The images of transverse eye sections stained with H&E were captured using slide scanner, as described above. Cell nuclei in the major retinal layers (ONL, INL, and GCL) were quantified using the Stardist deep learning platform with Fiji-ImageJ. First, the mrxs files were converted into tiff format. The Stardist Versatile (“*H&E Nuclei*”) pre-trained model was used in the analysis. The model was originally trained on images from the MoNuSeg 2018 training data and the TCGA archive [37]. For all retinal layers, images were normalized and the lower and upper percentiles were selected as 1 and 99.8, respectively. For GCL, probability/score threshold value was set to 0.5, and the overlap threshold was set to 0.4. For INL and ONL, the values were 0.1 and 0.6, respectively. After Stardist analysis, two representative 1000 µm long areas from both sides of the optic nerve head were chosen and the number of nuclei per area unit was calculated. The drawing of the regions started approximately 300 µm from the optic nerve head.

### TUNEL assay

Eye cryosections were fixed with 4% PFA for 15 min at RT. Then sections were treated with PBS containing 0.25% Triton X-100 and 3% BSA for 20 min at RT. To detect fragmented DNA in apoptotic cells within the retinal layers, “Click-iT™ Plus TUNEL Assay for In Situ Apoptosis Detection, Alexa Fluor™ 488 dye” (Invitrogen) was used in accordance with manufacturer’s protocol. A positive control sample was treated beforehand with 100 μg/mL of DNase-I (Sigma) to induce DNA strand breaks. After the TUNEL reaction, samples were mounted with ProLong^®^ medium with DAPI and imaged using Pannoramic Midi Fluoresence slide scanner (3DHistech Ltd., Budapest, Hungary). Apoptotic (TUNEL-positive) cells in the ganglion cell layer were counted manually using QuPath v.0.3.0 software [36]. Total ganglion cell numbers were analyzed using Stardist deep learning platform via Fiji-ImageJ [37]. The Versatile (fluorescent nuclei) model was used. Images were normalized, and lower percentile was set to 1.0 and upper percentile to 99.8. Probability threshold was 0.50 and Overlap threshold 0.40.

### Single cell analysis of mouse and human retina

We utilized publicly available single cell transcriptomic data of mouse retinal cells isolated from wild-type mice on a C57BL/6J background [38], Cx3cr1^YFP+^ myeloid cells isolated from retina of wild-type CB6F1/J mice [39], as well as human retinal cells isolated postmortem from cadaver eyeballs without apparent eye diseases [40], all generated with a 10 × chromium-based protocol. These data have been deposited in the National Center for Biotechnology Information (NCBI) Gene Expression Omnibus (GEO) database (https://www.ncbi.nlm.nih.gov/geo/) with accession numbers GSE132229, GSE126783 and GSE137537 for mouse retina, mouse Cx3cr1^YFP+^ microglial cells, and human retina scRNAseq data, respectively. The raw data were analyzed by Seurat (ver 4.0) for graph-based clustering and analysis of gene expression. Sctransform was applied to the data for normalization and variance stabilization of molecular count data (Hafemeister C, Genome Biology). Principal component analysis (PCA) was performed and a graph-based clustering approach was used by running the functions FindNeighbors and FindClusters. The clustering was visualized with Uniform Maniforld Approximation and Projection (UMAP). Cells were typed by examining expression of known marker genes. Markers used to phenotype cells in mouse retina included *Pde6a*, *Rho* (Rods), *Camk2b*, *Trpm1* (Bipolar cells), *Opn1sw*, *Opn1mw* (cones), *Glul* (Müller glia and astrocytes), *Gad1* (amacrine cells), *C1qa*, *Aif1* (microglia), *Pecam1* (endothelial cells), *Nefl* (retinal ganglion cells), *Onecut1*, *Onecut2*, *Lhx1* (horizontal cells), *Rgs5*, *Cspg4* (perivascular cells) [38]. Markers used to phenotype cells in mouse retinal Cx3cr1^YFP+^ cells included *P2ry12*, *P2ry13* (*P2ry12*^+^ microglia), *Hmox1*, *Ifrd1*, *Il1a* (*Hmox1*^+^ microglia), *Mrc1*, *Cxcl2* (perivascular macrophages), *Rorb*, *Rora* (*Rorb*^+^ macrophages) [39]. Markers used to phenotype cells in human retina included PDE6A, RHO (rods), GLUL (Müller glia and astrocytes), NEFL (retinal ganglion cells), CAMK2B, TRPM1 (bipolar cells), GAD1 (amacrine cells), GNAT2, OPN1SW, OPN1MW (cones), ONECUT1, ONECUT2, LHX1 (horizontal cells), C1QA, AIF1 (microglia) and PECAM1 (endothelial cells) [40].

### Statistical analysis

Statistical significance was determined by using two-tailed Student’s *t* test and Mann–Whitney *U* test. In the case of fERG study, the difference between the control and treated groups was evaluated by multiple *t* test grouped analysis using the Holm–Sidak method. The levels of statistical significance were denoted as *P < 0.05 and **P < 0.01. For competitive analysis, concentration–inhibition curves were generated in three to five separate experiments, and the IC_50_ values were calculated from one-site competition curves constructed using nonlinear least-squares curve fitting. All results were analyzed with Prism GraphPad 7 software (GraphPad, San Diego, CA, USA).

## Results

### CD73 is selectively compartmentalized in the photoreceptor layer of the mouse retina, while CD39 is highly expressed in the eye vasculature, retinal microglia and cornea

**Fig. 1:**
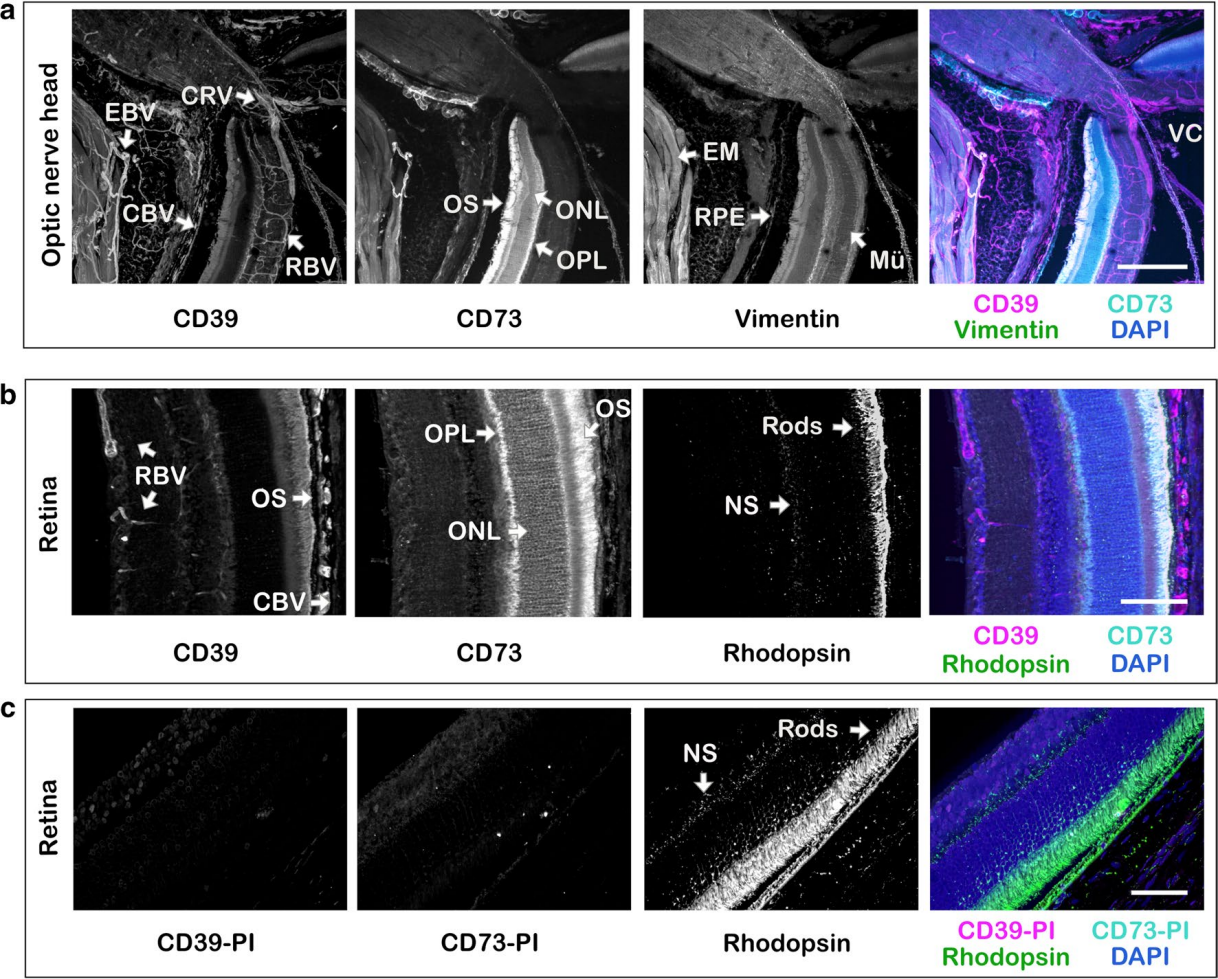
Immunofluorescence analysis of the distribution of CD73 and CD39 in the mouse retina and optic nerve head. LMA-embedded sections of the mouse eye were stained in free-floating assays with rabbit anti-CD73 (rNu9_L_-I5) and guinea pig anti-CD39 (mN1-2_C_I5) antibodies, together with molecular markers of the intermediate filaments of Müller cells (vimentin) and rod photoreceptor cells (rhodopsin), as indicated. The 3D images of the laminar region of the optic nerve head (**a**) and the medial regions of retina (**b**) were captured using a spinning disk confocal microscope. **c** In the negative control staining the eye sections were incubated with CD39-PI (mN1-2_C_PI) and CD73-PI (rNu9L-PI) pre-immune sera used at the same dilutions as in the **b** staining with primary anti-CD39 and anti-CD73 antibodies. Maximum intensity projections for each channel are shown in grayscale, with the right panels displaying merged images with nuclei counterstained with DAPI. *CBV* choroid blood vessels, *CRV* central retinal vessels, *EBV* extraocular blood vessels, *EM* extraocular muscles, *Mü* Müller cells, *NS* nonspecific staining (caused by binding of mouse anti-mouse rhodopsin antibody to endogenous immunoglobulins in the blood vessel), *ONL* outer nuclear layer, *OPL* outer plexiform layer, *OS* outer segments of photoreceptors, *PI* pre-immune serum, *VC* vitreous cavity. Scale bars: 300 μm (**a**), 100 μm (**b**), and 50 μm (**c**)

**Fig. 2:**
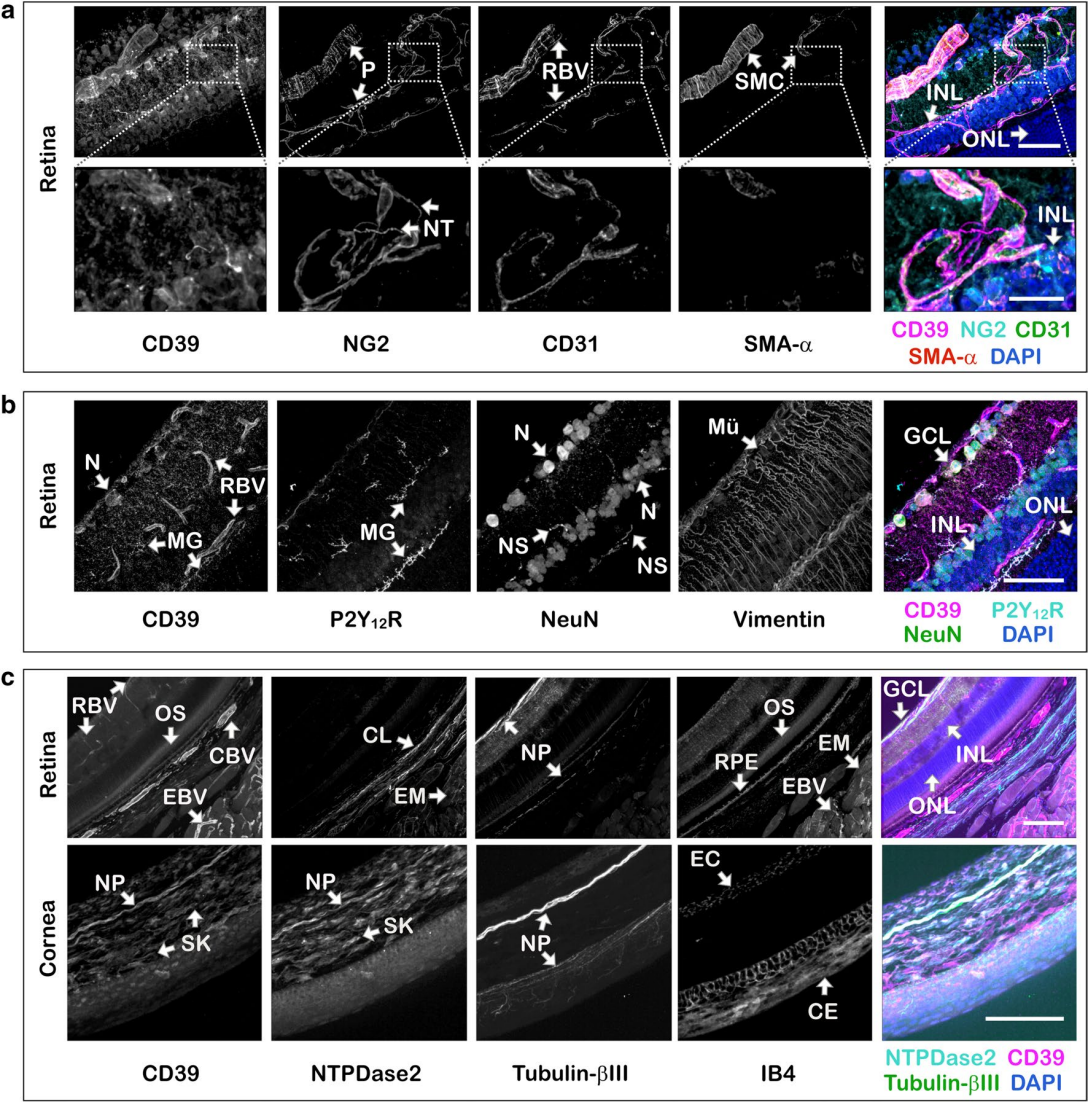
Multiplexed analysis of the distribution of CD39 and NTPDase2 in the mouse retina and cornea. LMA-embedded sections of the mouse eye were stained with antibodies against NTPDase1/CD39 and NTPDase2, together with molecular markers for pericytes (NG2), microglial cells (P2Y_12_R) neuronal bodies (NeuN), neuronal filaments (tubulin-βIII), smooth muscle cells (SMA-α), and the intermediate filaments of Müller glial cells (vimentin), as indicated. The 3D images of the medial region of retina and cornea were captured using a spinning disk confocal microscope. Maximum intensity projections of a confocal z-stack for each channel are shown in grayscale. The right-hand panels display merged images of selected channels with nuclei counterstained with DAPI. The arrows in the lower inset to **a** denote fine structures of NG2^+^ interpericyte tunneling nanotubes (NT) that connect two bona fide pericytes on separate capillary systems in the intermediate plexus. *CBV* choroidal blood vessels (choriocapillaris), *CE* corneal epithelium, *CL* choroidal layer, *EBV* extraocular blood vessels, *EC* endothelial cells, *EM* extraocular muscles, *GCL* ganglion cell layer, *INL* inner nuclear layer, *MG* microglial cells, *Mü* Müller cells, *N* neuronal body, *NP* neuronal processes, *NS* nonspecific staining (caused by binding of mouse anti-mouse NeuN antibody to endogenous immunoglobulins in the blood vessel), *ONL* outer nuclear layer, *OS* outer segments of photoreceptors, *RBV* retinal blood vessels, *RPE* retinal pigmented epithelium, *SK* stromal keratocytes, *SMC* smooth muscle cells. Scale bars: 50 µm (upper panels in **a** and **c**, **b**), 20 μm (lower panels in **a** and **c**)

The first part of this study was designed to assess the tissue-specific distribution of nucleotide-inactivating enzymes in the naïve mouse eye. The use of sections of whole eyeball dissected from C57BL/6N mice and embedded in low melting point agarose (LMA) and their sequential incubation with antibodies against CD73, CD39 and NTPDase2 in combination with a wide range of molecular markers allowed us to characterize the phenotypic identity and spatial localization of key ecto-nucleotidases in a relatively thick (~ 100 μm) tissue volume. Staining of the eye with anti-CD73 antibody (Fig. 1a, b), but not with isotype-matched rabbit pre-immune serum (Fig. 1c), revealed selective compartmentalization of CD73 in the photoreceptor layer. The highest CD73 immunoreactivity was associated with the outer segments (OS) of photoreceptor cells, where it is co-localized with a light-sensitive receptor protein rhodopsin (a marker of rod cells) (Fig. 1b). Another nucleotide-inactivating enzyme CD39 is highly expressed in the retinal vasculature, including the central retinal artery and vein, which enter the optic nerve head and further bifurcate into smaller arterioles, venules and capillaries extensively branching throughout the inner (superficial) plexus and deeper capillary plexus, as well as in the choroid layer (choriocapillaris) and extraocular blood vessels (Fig. 1a). Co-staining of the eyes with anti-CD39 antibody and different vascular markers demonstrated the presence of CD39 on all components of the vessel wall, including CD31^+^/IB4^+^ vascular endothelial cells which share their basement membranes with adjacent NG2^+^/Phalloidin^+^ pericytes, and also contractile SMA-α^+^/Phalloidin^+^ smooth muscle cells (SMC) wrapped in a circumferential pattern around larger arterioles (Fig. 2a and Fig. S1). Interestingly, the close-up view of the deep and intermediate plexuses of the mouse retina validated recent data on the presence of so-called “interpericyte tunnelling nanotubes” that connect two bona fide pericytes on separate capillary systems and regulate neurovascular coupling in the living retina [41], and further extend these observations by showing that these fine structures do not express CD39 and as a consequence are unable to metabolize ATP (Fig. 2a, inset). CD39 is also expressed, albeit faintly compared to blood vessels, on other ocular structures, including rhodopsin^+^ OS of photoreceptor cells (Fig. 1b), NeuN^+^ neuronal cell bodies located in the ganglion cell layer (GCL), as well as P2Y_12_R^+^ microglial cells, which mainly reside in two synaptic compartments of the neural parenchyma: the outer plexiform layer (OPL) and the inner plexiform layer (IPL), and in the optic nerve head (Fig. 2b). Furthermore, CD39 is co-localized with another member of the NTPDase family, NTPDase2, on tubulin-βIII^+^ neuronal processes lining the innermost margin of the retina and cornea, as well as corneal IB4^+^/Phalloidin^+^ epithelial cells, and stromal keratocytes (Fig. 2c and Fig. S1). The specificity of CD39 staining was further confirmed by the absence of any fluorescence signal in the negative control sample incubated with guinea pig pre-immune serum used at the same dilution as the primary anti-CD39 antibody (Fig. 1c).

**Fig. 3:**
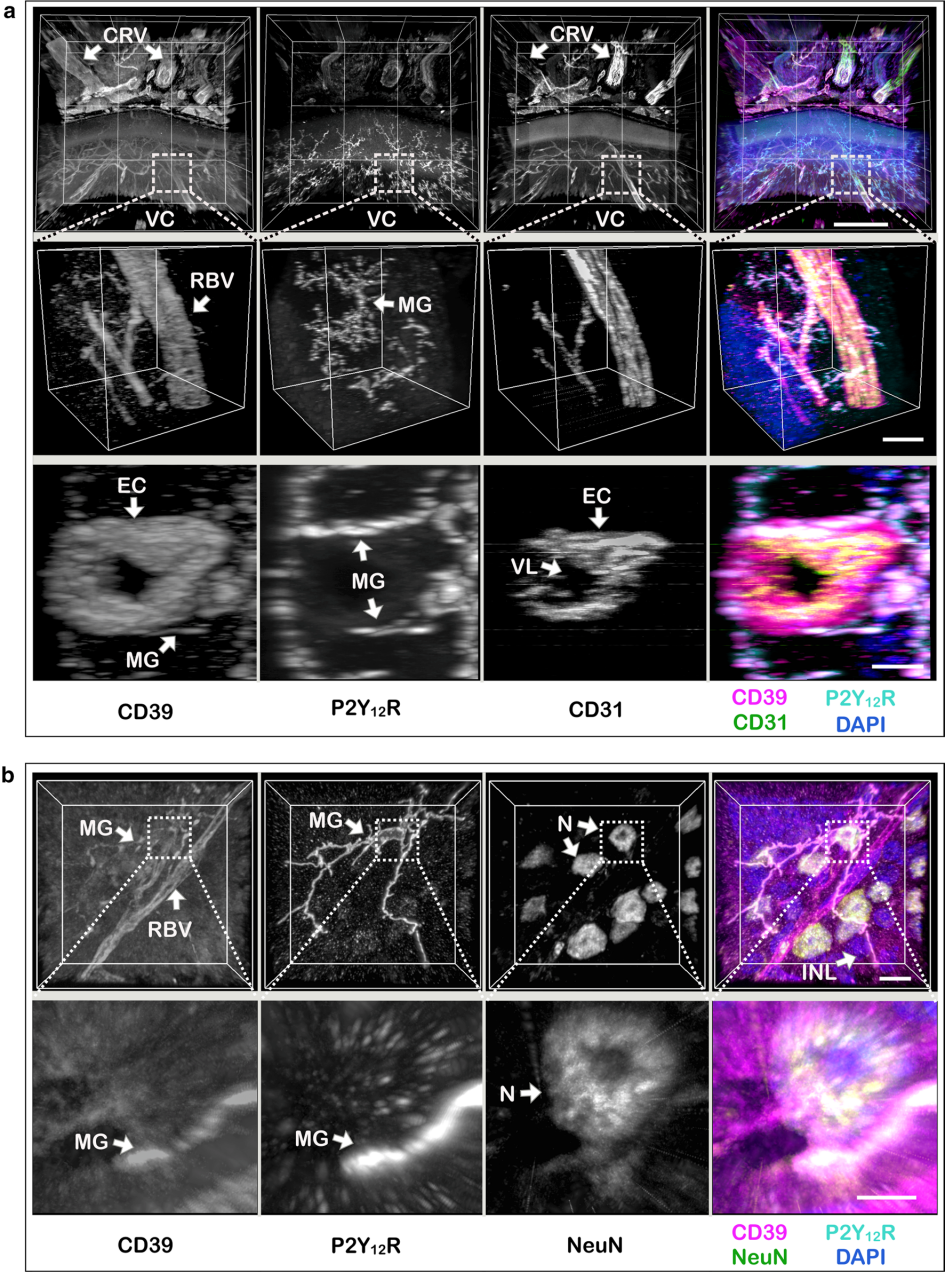
3D imaging identifies specific “purinergic junctions” in the mouse eye formed via direct interactions between microglial processes, retinal blood vessels and neuronal cell bodies. LMA-embedded sections of the mouse eye were co-stained with anti-CD39 antibody and molecular markers of blood endothelial cells (CD31), microglial cells (P2Y_12_R), and neuronal cell bodies (NeuN), as indicated. Z-stacks of the medial retina were captured using a spinning disk confocal microscope, and presented as reconstructed 3D images. Single channels are shown in grayscale and the right-hand panels display merged images with nuclei counterstained with DAPI. The insets display 3D images of representative areas at a higher magnification. The bottom inset of **a** shows a close-up of retinal arteriole, cropped and rotated 90° to visualize the lumen of the vessel. *CRV* central retinal vessels, *EC* endothelial cells, *INL* inner nuclear layer, *MG* microglia, *N* neuron, *RBV* retinal blood vessels, *VC* vitreous cavity, *VL* vessel lumen. Scale bars: 200 μm (**a**), 30 μm (upper inset in **a**), 10 μm (**b**, bottom inset in **a**), and 2 μm (inset in **b**). 3D reconstructed datasets from **a** and **b** were also rendered as movie sequences and are presented in Supplementary data as Movie 1.mp4 and Movie 2.mp4, respectively

The advantage of our workflow is that it provides additional information on high-resolution 3D mapping of cell positioning in the context of macroscale tissue. Given that the commonly used 2D immunofluorescence images or maximum intensity projections of 3D images significantly underestimate microglial cell motility [31, 42], such volumetric approach may be particularly relevant for evaluation of stereoscopic morphology of retinal microglial processes and their heterotypic interactions with other components of the neurovascular unit. The 3D reconstructed images enabled visualisation of extensively branched microglial cell processes that co-express two important components of the purinergic machinery, P2Y_12_R and CD39, and form direct contacts with exterior walls of CD39^+^ retinal blood vessels (Fig. 3a, and Movie 1), as well as with neuronal cell bodies which express CD39 at relatively low levels (Fig. 3b, and Movie 2).

### In situ enzyme histochemistry and flow cytometric assays confirm cell type- and tissue-specific localization of ecto-nucleotidases in the mouse retina

**Fig. 4:**
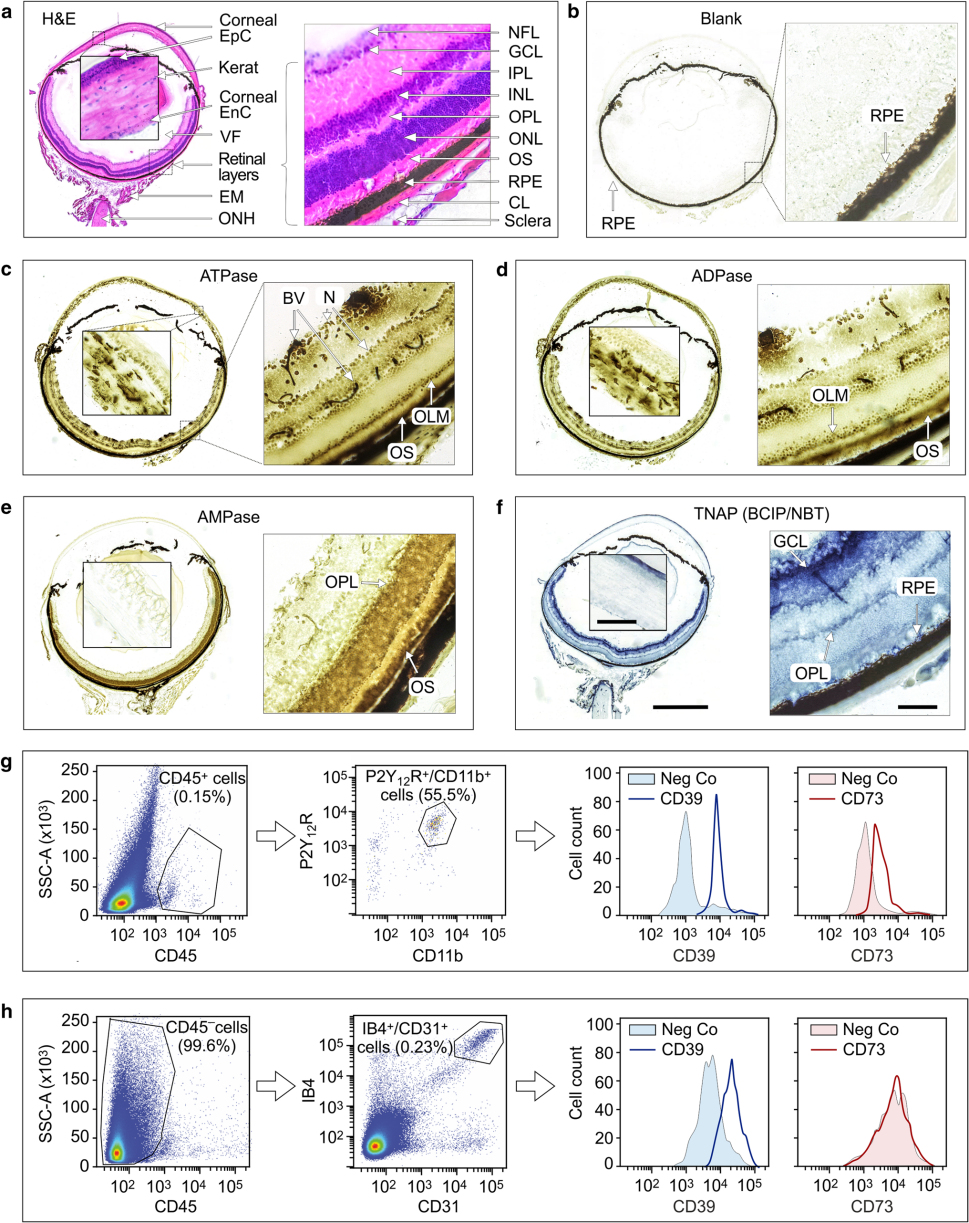
In situ enzyme histochemistry and flow cytometric analysis of the expression of ecto-nucleotidases in the mouse eye. **a** Eyeballs were enucleated from C57BL/6N mice and major retinal layers and other ocular structures were visualized by hematoxylin and eosin (H&E) staining. **b**–**e** Ecto-nucleotidase activities were assayed in situ by incubating eye cryosections for 30 min with Pb(NO_3_)_2_ in the absence (“Blank”, **b**) and presence of ATP (**c**), ADP (**d**), and AMP (**e**) followed by microscopic detection of the nucleotide-derived inorganic phosphate (P_i_) as a brown precipitate. **f** The activity of tissue-nonspecific alkaline phosphatase (TNAP) was measured by using the artificial chromogenic substrates BCIP and NBT and subsequent monitoring of the development of the blue color reaction. All images were captured by using Pannoramic 250 slide scanner. *BV* blood vessels, *EM* extraocular muscles, *EnC* endothelial cells, *EpC* epithelial cells, *GCL* ganglion cell layer, *IPL* inner plexiform layer, *INL* inner nuclear layer, *N* neurons, *NFL* nerve fiber layer, *OLM* outer limiting membrane, *ONH* optic nerve head, *ONL* outer nuclear layer, *OPL* outer plexiform layer, *OS* outer segments of photoreceptor cells, *RPE* retinal pigmented epithelium, *VF* vitreous fluid. Scale bars: 1 mm (left) and 80 μm (“inside the eyeball” and right-hand insets). **g**,** h** Flow cytometric analysis of CD39 and CD73 expression in the mouse retina. Single-cell suspension of freshly isolated retinal cells was incubated with anti-CD73 and anti-CD39 antibodies, together with fluorescently-labeled antibodies against CD45, CD11b, P2Y_12_R, and CD31, and also biotin-conjugated Isolectin B4 (IB4), as indicated. The right-hand panels show histograms of CD45^+^/P2Y_12_R^+^/CD11b^+^ microglial cells (**g**) and CD45^−^/IB4^+^/CD31^+^ vascular endothelial cells (**h**) stained with anti-CD39 and anti-CD73 antibodies, as well as isotype-matched control immunoglobulins (*Neg Co*). Data are representative of three independent experiments

In a different set of experiments, the activities of ecto-nucleotidases were measured in the mouse eye cryosections by using lead nitrate-based enzyme histochemistry assay [34]. Additional staining of the samples with haematoxylin and eosin (H&E) (Fig. 4a) enabled the visualization of the main retinal layers and other ocular structures. The presence of dark light-absorbing melanin granules in the exterior retinal pigmented epithelium (RPE) partially interferes with enzyme histochemistry of the eye. Nevertheless, there were clear-cut differences in staining intensities between the samples incubated without (Fig. 4b) and with (Fig. 4c–e) exogenous nucleotides. High ATPase (Fig. 4c) and ADPase (Fig. 4d) activities were detected in the retinal vessels, OS of photoreceptor cells, outer limiting membrane, and neuronal bodies, while AMP-specific staining was mainly confined within the photoreceptor layer (Fig. 4e). High ATPase and ADPase (but not AMPase) activities were also detected in the stromal keratocytes and basal epithelial layer of the cornea (Fig. 4c–e). Notably, similar staining patterns were observed when eye cryosections were incubated with nucleotide substrates in the presence (Fig. 4c–e) and absence (data not shown) of the inhibitor of tissue-nonspecific alkaline phosphatase (TNAP) tetramisole. On the other hand, the use of the artificial chromogenic substrates of TNAP, BCIP and NBT revealed the development of specific dark blue staining in the inner and outer plexiform layers of retina, as well as in the superficial corneal epithelial cell layer (Fig. 4f), which disappeared after pretreating the samples with tetramisole (data not shown). These data suggest that despite the selective expression of TNAP in certain eye structures, this broad substrate-specificity ectoenzyme is not implicated in the metabolism of ocular ATP and other nucleotides. Collectively, in situ enzyme histochemistry, together with the multiplexed imaging data described above, identified CD39 as the predominant ATP- and ADP-inactivating enzyme in the mouse eye which is expressed to varying degrees among vascular, immune, neural and stromal cells. The downstream step of hydrolysis of ATP/ADP-derived AMP into ADO is mediated through ecto-5′-nucleotidase/CD73 activity, which is mainly localized in the photoreceptor layer. Flow cytometric analysis of isolated mouse retinal cells provided independent line of evidence for the presence of CD39 on CD45^+^/CD11b^+^/P2Y_12_R^+^ microglial cells (Fig. 4g) and CD45^−^/IB4^+^/CD31^+^ vascular endothelial cells (Fig. 4h). CD73 is also weakly expressed on retinal microglial cells, but not in the blood vessels (Fig. 4g, h).

### Single cell transcriptomic analysis of mouse and human retinal cells reveals relatively conserved purinergic signatures between the species

**Fig. 5:**
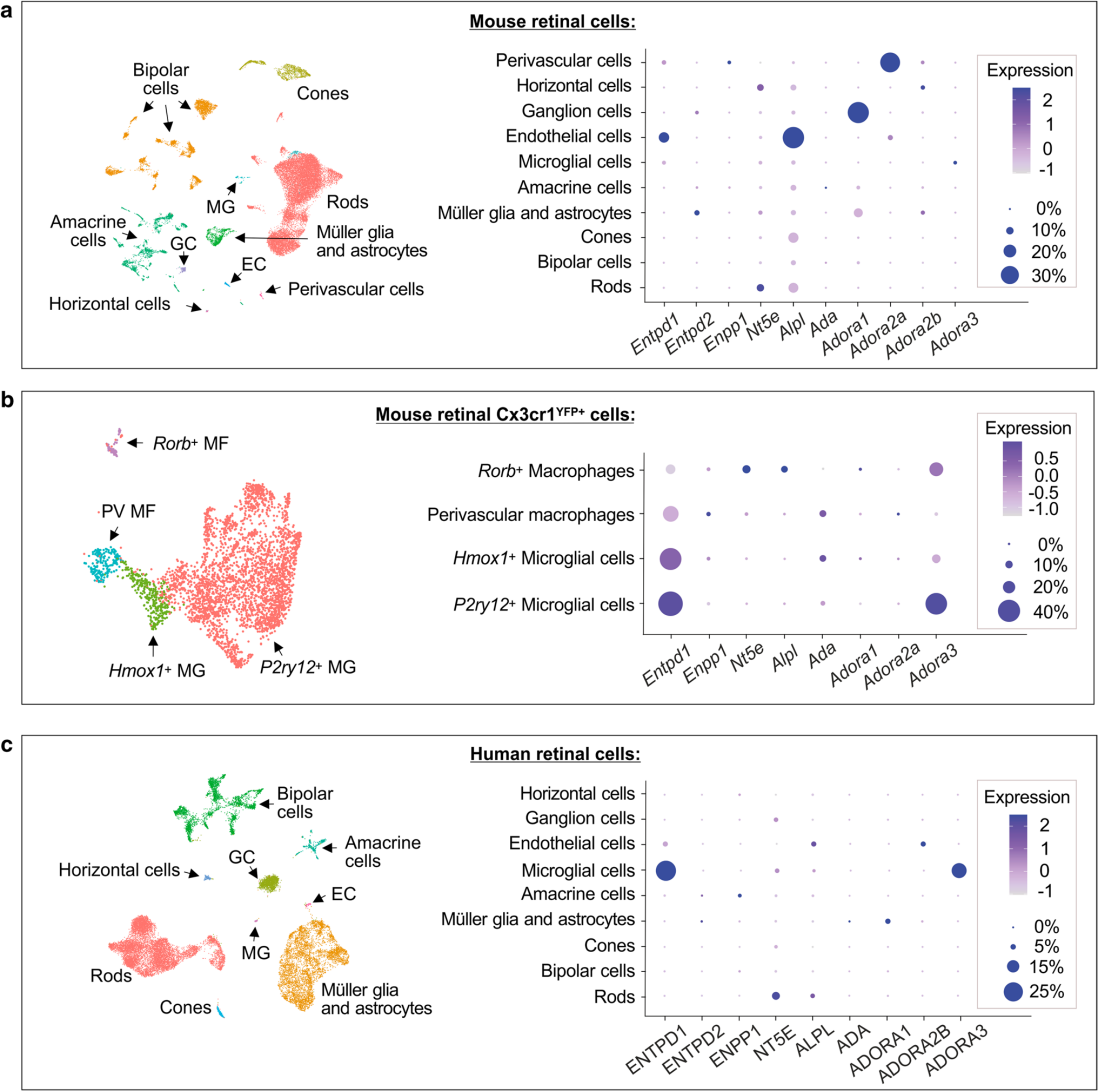
Single cell transcriptomic analysis of purine-inactivating enzymes and ARs in the mouse and human retinal cells. Single cell RNA sequencing (scRNAseq) analysis of mouse retinal cells from Heng et al. [38] (**a**), mouse retinal Cx3cr1^YFP+^ cells from O’Koren et al. [39] (**b**), and human retinal cells from Menon et al. [40] (**c**). Left panels show uniform manifold approximation and projection (UMAP) of cell types in the retina. Known markers of retinal cells were used to identify each cell type (see also “Materials and methods”). Dot plots (right panels) show the expression of key enzymes of ADO metabolism, NTPDase1/CD39 (mouse and human gene names *Entpd1* and ENTPD1, respectively), NTPDase2 (*Entpd2* and ENTPD2), ecto-nucleotide pyrophosphatase/phosphodiesterase-1 (*Enpp1* and ENPP1), ecto-5′-nucleotidase/CD73 (*Nt5e* and NT5E), TNAP (*Alpl* and ALPL), adenosine deaminase (*Ada* and ADA), as well as ADO receptor subtypes A_1_R (*Adora1* and ADORA1), A*2A*R (*Adora2a*), A_2B_R (*Adora2b* and ADORA2B) and A_3_R (*Adora3* and ADORA3) in mouse (**a**, **b**) and human (**c**) retinal cell subsets identified in UMAP plots. The relative expression levels of the indicated genes are shown on a pseudocolor scale (log2(FC)), with the size of the dot representing the percentage of cells in a subset where the gene is detected. Markers used to phenotype different subsets of mouse retinal Cx3cr1^YFP+^ cells included *P2ry12*, *P2ry13* (*P2ry12*^+^ microglia), *Hmox1*, *Ifrd1*, *Il1a* (*Hmox1*^+^ microglia), *Mrc1*, *Cxcl2* (perivascular macrophages), *Rorb*, *Rora* (*Rorb*^+^ macrophages). NTPDase2 (*Entpd2*) and A_2B_R (*Adora2b*) were not found in the mouse retinal microglial cells (**b**), and A_2A_R (ADORA2A) was not found in the human dataset (**c**)

The expression profiles of genes encoding major purine-inactivating enzymes and ARs were also characterized at a single cell resolution by using publicly available scRNAseq data of mouse retinal cells [38]. Single cell transcriptomic analysis demonstrated specific distribution of ectoenzymes in mouse vascular endothelial cells (*Entpd1*/CD39^high^, *Alpl*/TNAP^high^), perivascular cells (*Entpd1*/CD39^low^, *Enpp1*/ENPP1^low^), retinal microglia (*Entpd1*/CD39^low^), rod photoreceptors (*Nt5e*/CD73^high^, *Alpl*/TNAP^low^), horizontal cells (*Nt5e*/CD73^high^), RGC (*Entpd2*/NTPDase2^low^), Müller glia and astrocytes (*Entpd2*/NTPDase2^high^) (Fig. 5a). In contrast to our multiplex imaging data showing the presence of CD39 immunoreactivity (Fig. 1b) and ATP/ADP-inactivating activity (Fig. 4c, d) in the OS of photoreceptor cells, transcriptomic approach did not reveal CD39-encoding gene in rod cells at mRNA level (Fig. 5a). Although the expression of *Entpd1* on microglial cells was very low in this study, the use of another scRNAseq dataset of sorted Cx3cr1^+^ mouse retinal cells [39] revealed that *Entpd1*/CD39 is highly expressed on two major populations of *P2ry12*^+^ and *Hmox1*^+^ microglial cells, and additionally demonstrated the presence of other enzyme of the purine catabolic chain, ADA, on retinal *Hmox1*^+^ microglial cells and perivascular macrophages (Fig. 5b). While a detailed characterization of signal transduction pathways mediating biological effects of ADO lies beyond the scope of this study, we also analyzed the expression profiles of major AR subtypes. ARs are selectively expressed on various mouse retinal cells, including vascular endothelial (*Adora2a*/A_2A_R^low^) and perivascular (*Adora2a*/A_2A_R^high^) cells, RGC (*Adora1*/A_1_R^high^), Müller glia and astrocytes (*Adora1*/A_1_R^low^), *P2ry12*^+^ microglial cells and *Rorb*^+^ macrophages (*Adora3*/A_3_R^high^) (Fig. 5a, b). Notably, data on highly selective expression of *Nt5e*/CD73 on the latter subset of *Adora3*^+^*/Rorb*^+^ macrophages suggest that ADO metabolism may be relevant in controlling adenosinergic signaling and function in this relatively small population of mouse retinal myeloid cells (Fig. 5b).

To further identify the similarities and differences in the purinergic signatures between rodent and human eyes, we utilized single cell transcriptomic atlas of the human retina [40]. Ecto-nucleotidases and TNAP are selectively expressed on the human retinal endothelial cells (ENTPD1/CD39^low^, ALPL/TNAP^high^), microglial cells (ENTPD1/CD39^high^, NT5E/CD73^low^), rod photoreceptors (NT5E/CD73^high^, ALPL/TNAP^low^), amacrine cells (ENPP1/ENPP1^low^), and Müller glia and astrocytes (ENTPD2/NTPDase2^low^), while the expression of ADO-inactivating enzyme ADA was maintained at very low or undetectable levels in all human retinal cells (Fig. 5c). These human transcriptomic data are consistent with our recent in situ enzyme histochemistry and immunofluorescence imaging data showing tissue-specific distribution of key ecto-nucleotidases (CD39, NTPDase2, CD73) and TNAP in the human sensory neuroretina and optic nerve head [16]. Additional scRNAseq analysis of adenosinergic signaling pathways revealed that, similar to mouse retina, human retinal microglial cells and Müller glia and astrocytes express high levels of A_3_R (ADORA3) and A_1_R (ADORA1), respectively. However, unlike mouse blood vessels which express *Adora2a*/A_2A_R, human retinal endothelial cells express another A_2_R subtype, ADORA2B/A_2B_R. Other human retinal cell subsets do not appear to express either AR subtype (Fig. 5c).

Overall, despite some species-specific variations, the expression profiles of key purinergic enzymes and ARs appear to remain relatively conserved between the mouse and human eyes. In particular, data on selective compartmentalization of CD73 in both mouse and human photoreceptors provide a solid background for more thorough investigation of the role of this ectoenzyme in retinal function under various challenging and noxious conditions and further translation of these experimental data to clinic.

### Pharmacological inhibition of ocular CD73 impairs retinal activity in dark-adapted mice exposed to bright light

**Fig. 6:**
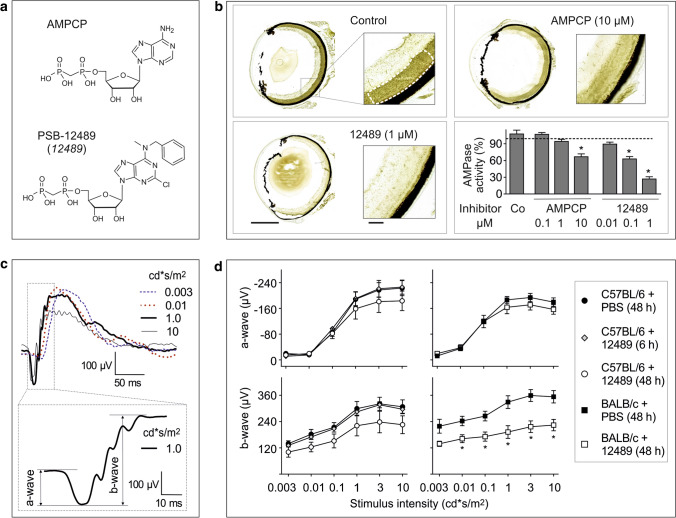
The effect of CD73 inhibitors on AMPase activity and electrical activity of the mouse retina. **a** Chemical structures of the standard CD73 inhibitor, AMPCP, and the new inhibitor PSB-12489 (*12489*). **b** The effect of CD73 inhibitors on retinal AMPase activity was determined in situ by incubating mouse eye cryosections with 1 mM AMP and 2 mM Pb(NO_3_)_2_ in the absence (control) and presence of the indicated concentrations of inhibitory compounds. Mean pixel intensities of AMP-specific brown staining were quantified in the selected regions of photoreceptor layer using QuPath v.0.2.0 software, and expressed as a percentage of control activity (mean ± SEM; *n* = 3). *P < 0.05 compared with control, determined by Student’s *t* test (paired, two-tailed). **c** Electrical activity of retina was examined in live animals using fERG. The upper panel shows representative fERG waveforms recorded from scotopic retinas stimulated at light intensity increments from 0.003 to 10 cd s/m^2^. The amplitude of the a-wave was measured from the baseline to the lowest point of the wave, while the b-wave was measured from the trough of the a-wave to the highest point, as indicated in the lower inset. **d** PSB-12489 (*12489*) and PBS were injected into the vitreous cavity of C57BL/6JrJ (*C57BL/6*) and BALB/c mice. fERG responses were measured in the dark-adapted eyes 6 or 48 h after the treatment. The graphs show the amplitudes of the a-waves (upper panels) and b-waves (lower panels) versus luminance intensity (mean ± SEM; n = 4). *P < 0.05, determined by multiple *t* test grouped analysis

Taking into account data on direct involvement of adenosinergic signaling in the modulation of light-evoked responses of retina [43–45], we hypothesized that pharmacological inhibition of the CD73–ADO axis may affect retinal function. Several novel CD73 inhibitors have been designed and synthesized recently in our laboratories based on *N*^6^-benzyl-α,β-methylene-ADP (PSB-12379) as a lead structure, which are characterized by exceptionally high selectivity, nanomolar inhibitory potency toward human, rat and mouse CD73, and high metabolic stability in human plasma and in rat liver microsomes [27]. Studies with fluorescein-conjugated CD73 inhibitors additionally confirmed the utility of these compounds as fluorescent probes capable of binding directly to CD73 on various cells and tissues, including mouse CD73^+^ photoreceptor cells [46]. The most potent CD73 inhibitor, PSB-12489 (Fig. 6a) [27], was chosen as a suitable drug for further examination in our competitive and functional assays. Radio-TLC enzymatic assays confirmed the ability of PSB-12489 to inhibit the hydrolysis of [^3^H]AMP by human and mouse sera in a concentration-dependent manner with the IC_50_ values in the low nanomolar range, whereas the classical CD73 inhibitor AMPCP exerted inhibitory effects at ~ 100 times higher concentrations (Fig. S2). This conclusion was independently ascertained by in situ enzyme histochemistry showing that treatment of mouse eye cryosections with increasing concentrations of PSB-12489 (0.1–1 μM), but not with equimolar concentrations of AMPCP, progressively reduced the intensity of AMP-specific staining in the photoreceptor layer (Fig. 6b).

The effect of CD73 inhibitor on the retinal function was assessed in vivo. Electrophysiological analysis of the retina was performed by recording fERG responses from dark-adapted (scotopic) eyes stimulated with increments of light intensity from 0.003 to 10 cd s/m^2^. Figure 6c shows representative electroretinograms, which can be divided into the following components: the first a-wave that appears as a negative amplitude change, and the b-wave that appears as a large positive amplitude change immediately after the a-wave. The a-wave of the electroretinogram reflects the functional activity and integrity of the photoreceptors, whereas the b-wave originates in retinal cells that are post-synaptic to the photoreceptors, including inner retinal cells (bipolar and amacrine cells) and RGC [43, 47, 48]. Notably, C57BL/6N mice are known to be homozygous for the rd8 mutation in Crumbs homolog 1 (*Crb1*) gene, which may lead to severe retinal dysplasia in the inferior retina and other ocular abnormalities [49]. These lesions appear as white to yellow flecks on fundus examination, and the phenotype is worsened by exposure of C57BL/6N mice to BL [50]. Therefore, we first compared the effects of CD73 inhibitor on retinal electrical activity in different strains of mice. To achieve sufficient inhibitory effect, PSB-12489 was injected locally into the vitreous cavity at a relatively high dose, with a final concentration of ~ 200 µM in the eye. Measurement of fERG responses in C57BL/6JrJ mice showed no differences in a-wave and b-wave amplitudes between the groups that received CD73 inhibitor or PBS for 6 or 48 h. On the other hand, treatment of BALB/c mice with PSB-12489 for 48 h was accompanied by significant decreases in the b-wave amplitudes recorded at light intensities from 0.01 to 1 cd s/m^2^ (Fig. 6d). Based on these observations, BALB/c mice were chosen as an appropriate model for further investigation of the role of ADO metabolism in retinal function and BL-induced phototoxicity.

**Fig. 7:**
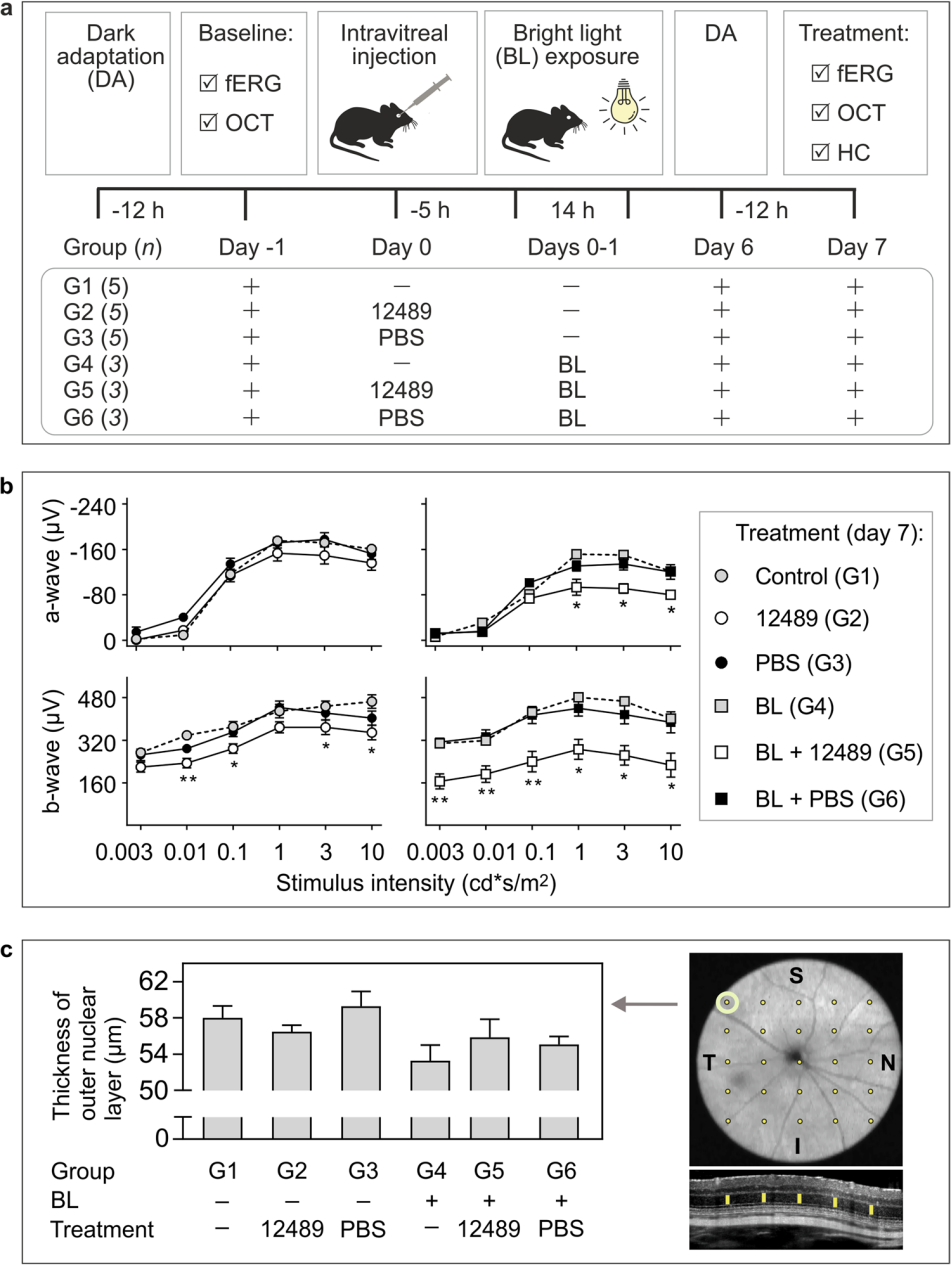
Inhibition of CD73 in dark-adapted mice before exposure to bright light impairs fERG responses, but has no effect on total retinal thickness. **a** Experimental design for analysis of the effects of CD73 inhibitor on the retinal function and structure. BALB/c mice were divided into six groups, which either remained untreated (G1 and G4) or received a single bilateral injection of PSB-12489 (*12489*; G2 and G5) and vehicle (*PBS*; G3 and G6). The animals were kept in transparent plastic cages without any treatment (G1–G3) or additionally exposed to continuous bright light (*BL*; G4–G6). Electrical responses of the retina and retinal thicknesses were examined in live mice both at baseline and at the end of the experiment. **b** fERG responses were measured in the dark-adapted eyes 7 days after the treatments. The graphs show the amplitudes of the a-waves (upper panels) and b-waves (lowe panels) versus luminance intensity (mean ± SEM; *n* = 6–10). *P < 0.05 and **P < 0.01, determined by multiple *t* test grouped analysis. **c** The thickness of outer nuclear layer was determined by high-resolution spectral domain optical coherence tomography. The right-hand image depicts retina fundus with superior (S), inferior (I), temporal (T), and nasal (N) parts of the retina. Twenty-five spots were selected for retinal thickness analysis, with the central point targeted at the centre of the optic nerve. Total retinal thickness was measured from inner plexiform layer to external limiting membrane, as shown in the lower cross-sectional image of retina. The left panel displays the outer nuclear layer thickness determined in the most superior temporal area of the retina (top left cell) of the treated mice (mean ± SEM; *n* = 6).

To study the role of CD73-generated ADO in the maintainance of retinal activity, BALB/c mice remained untreated or received a single intravitreal injection of PSB-12489 or vehicle (PBS), and subsequently exposed to continuous illumination for 14 h, as schematically illustrated in Fig. 7a. Electrical activity of retina was examined in live animals at baseline and 7 days after the treatment. Measurement of basal fERG values before treatment did not detect any differences in the a-wave and b-wave amplitudes in the study groups (Fig. S3a). However, when fERG was repeated on day 7 post-treatment, relatively moderate but significant decreases in the b-wave amplitudes were found in the PSB-12489-treated eyes (group G2), when compared to vehicle-treated (G3) and non-treated control (G1) groups (Fig. 7b). These differences became even more substantial after exposing the PSB-12489-treated animals to BL (group G5). These mice were characterized by ~ 40–50% decrease in the b-wave amplitudes at all stimulus levels of the light intensity tested and also showed a decrease in the a-wave amplitude recorded at high light intensities (1–10 cd s/m^2^), when compared to vehicle-treated (G6) and non-treated (G4) groups exposed to BL (Fig. 7c). Notably, in contrast to the conventional experimental model of BL-induced retinal damage induced by extending the period of dark adaptation up to 24 h and characterized by markedly impaired scotopic responses (our unpublished observations), the combination of dark adaptation and light illumination parameters used in this work did not by itself cause any adverse effects on retinal electrical activity (Fig. S3b).

### Impaired functional responses of retinal cells in PSB-12489-treated mice were not accompanied by decrease in total thickness of the retina or death of retinal ganglion cells

The thickness of the retina was determined in live animals immediately after fERG recording by using high-resolution spectral domain optical coherence tomography (OCT). It was measured in superior temporal area of the retina, which is the most sensitive to the retinal damage. No significant changes in total retinal thickness were observed between the groups studied (Fig. 7c). The expression levels and activity of CD73 in the treated retina were also interrogated at the histological level. Given the uneven distribution of CD73 in the mouse photoreceptor layer, AMP-specific staining intensities were determined in three different regions, including highly CD73-positive OS of photoreceptor cells, as well as ONL and OPL characterized by intermediate enzyme expression (Fig. S4a). Quantitative analysis did not detect any down-regulation of AMPase activity in the eyes receiving PSB-12489 (Fig. S4b). This conclusion was independently ascertained by immunofluorescence assays showing a similar pattern of CD73 staining in rhodopsin^+^ photoreceptor cells in the control (Fig. S5a) and PSB-12489-treated (Fig. S5b) eyes. Notably, PSB-12489 acts as a reversible small-molecule inhibitor of CD73 and therefore, it can be washed out during preparation of eye cryosections and their subsequent incubation with exogenous AMP. Therefore, in situ enzyme histochemistry data on comparable AMPase activity in all groups do not rule out the possibility that PSB-12489 prevents intraocular adenosine production in live mice via temporal and reversible inhibition of CD73 throughout the whole period of treatment.

The numbers of nuclei in the retinal layers were quantified to provide a further assessment of cell survival in the treated eyes. Mice receiving PSB-12489 and exposed to BL did not show any significant changes in the total number of retinal neurons in the ONL, INL, or GCL (Fig. S6). To further assess potential harmful cytotoxic effects of the drug, we determined the number of apoptotic cells in the innermost retinal ganglion layer by using TUNEL Assay. Combined exposure of the mice to BL and PSB-1249 did not trigger any additional apoptosis in the treated retina, and even caused significant decrease in the number of TUNEL-positive RGC (Fig. S7).

## Discussion

**Fig. 8:**
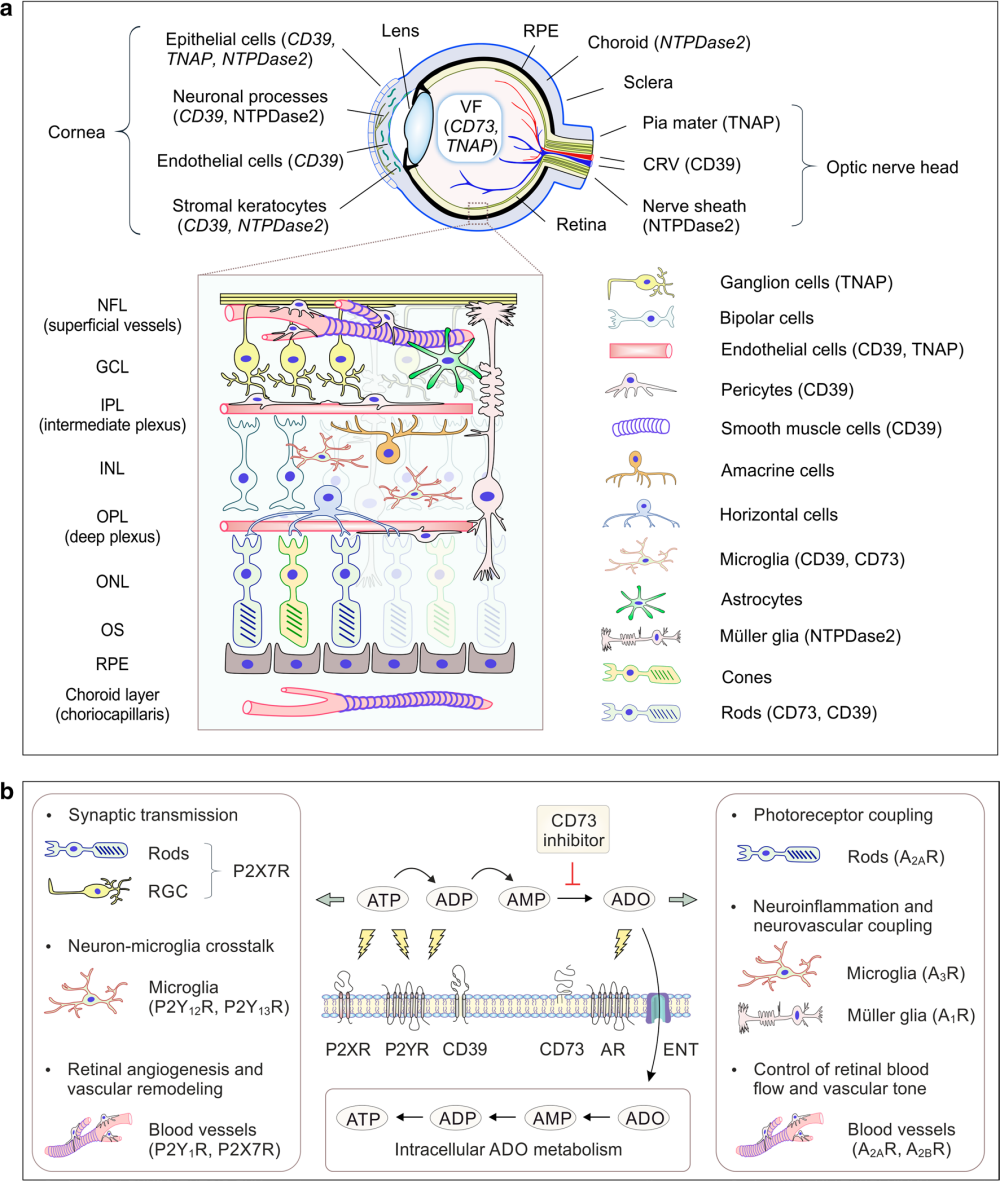
Schematic representation of the role of ADO metabolism in retinal functioning. **a** Schematic view of the distribution of ecto-nucleotidases in the mammalian eye. The upper cartoon illustrates the cross section of the eyeball and highlights the expression pattern of ecto-nucleotidases in the optic nerve head, cornea, and other ocular structures. The inset shows the cell-specific compartmentalization of ecto-nucleotidases in major retinal layers. *CRV* central retinal vessels, *GCL* ganglion cell layer, *NFL* nerve fiber layer, *IPL* inner plexiform layer, *INL* inner nuclear layer, *ONL* outer nuclear layer, *OPL* outer plexiform layer, *OS* outer segments of photoreceptor cells, *RPE* retinal pigmented epithelium. **b** Cellular purine turnover depends on interactions between extracellular ATP release, binding to nucleotide (P2YR and P2XR) and adenosine (AR) selective receptors, inactivation of nucleotides through ectoenzymes CD39 and CD73, cellular uptake of nucleotide-derived ADO via equilibrative nucleoside transporters (ENT) and its phosphorylation into intracellular ATP through complex phosphotransfer reactions. Site of directional inhibition of this metabolic cascade by selective CD73 inhibitor PSB-12489 is pointed by T-shaped arrow pointer. Potential mechanisms underlying the effects of CD73 inhibitor on retinal function can fall into three main categories: (i) the impaired activation of ARs due to the insufficient generation of ADO; (ii) a simultaneous shift in purine homeostasis from the generation of anti-inflammatory and vasoactive ADO toward a proinflammatory and cytotoxic ATP-regenerating phenotype; and (iii) a role that is linked to the reduced cellular uptake of ADO with subsequent deregulation of cellular bioenergetics and related signaling pathways

By investigating the combined features of ocular purine homeostasis and electrical activity of the retina, we have identified a link between these different but apparently interrelated processes. The major findings are summarized as follows: (i) this work identified the presence of an extensive and spatially arranged network of ectoenzymes in the mouse and human eyes where they coordinately control ATP and ADO levels; (ii) the role of the CD73-generated ADO was ascertained in functional in vivo assays showing that temporary inhibition of ocular CD73 activity in dark-adapted mice prior to their transition from darkness to light caused a decrease in fERG responses. These findings are summarized in Fig. 8 which schematically illustrates cell- and tissue-specific distribution of ecto-nucleotidases in the mammalian eye (panel a), and further highlights the role of the ATP–ADO axis in retinal functioning (panel b).

To our knowledge, this is the first study providing a holistic view of ocular purine metabolism and signaling as a complex and spatially integrated network. By using two independent and complementary approaches, in situ enzyme histochemistry and multiplexed imaging, we were able to pinpoint both the catalytic activities and the expression levels of major purinergic ectoenzymes in the mouse neuroretina, optic nerve head and cornea. These imaging data, in combination with dissociation-based flow cytometric and scRNAseq analyses of mouse and human retinal cells, provide sufficient justification for re-evaluating the existing models of ocular purine metabolism and its role in retinal functioning. Similar to ubiquitous expression of CD39 in the systemic circulation where it controls hemostasis through termination of prothrombotic, proinflammatory and vasoactive effects of circulating ATP and ADP [26, 51], CD39 was also shown to be highly expressed on blood vessels of various caliber located in the optic nerve head and retinal and choroid layers. On the other hand, contrary to the previous reports showing high CD73 expression on endothelial cells lining the lumen of large blood vessels, such as human and rodent aorta, carotid and coronary arteries [26, 52], and also central retinal vessels of human optic nerve head [16], we did not detect any CD73 immunoreactivity, AMPase activity, or *Nt5e*/CD73 gene expression in the mouse retinal vasculature. This observation contrasts with the current view of ADO as a key regulator of ocular blood flow and vascular tone which elicits its vasoactive effects through binding to A_2A_R/A_2B_Rs expressed on retinal endothelial and perivascular cells ([12], also Fig. 5). This apparent discrepancy can be explained by the existence of alternative pathways which ensure local ADO supply to the retinal vessels. These mechanisms might particularly include the direct release of endogenous ADO by vascular and neuronal cells via bidirectional equilibrative nucleoside transporters [29, 53], ADO formation at the vitreoretinal interface through soluble intravitreal CD73 activity [16, 23], as well as metabolism of AMP into ADO by neighboring CD73^+^ microglial cells located in close vicinity to the vessel wall (current study).

Microglia are the main resident macrophages in the central nervous system. They maintain brain homeostasis by monitoring and scavenging dying cells, engulfing synaptic material through a pruning process, and also responding to pathogenic stimuli by the release of IL-1β, TNF-α and other proinflammatory cytokines [31, 54, 55]. Recent studies have also demonstrated a key role for the ATP–ADO axis in microglia-driven inhibition of neuronal activity in mouse and human brains [54, 55], which mainly occurs via so-called “somatic microglia–neuron junctions” characterized by a highly specialized nanoarchitecture optimized for purinergic signaling [56]. While much of our knowledge concerning microglia–neuron interaction has been derived from brain research, these results are not directly transferrable to the retinal microglial cells which differ substantially in terms of morphological features and functional properties [57], and may also undergo dramatic transcriptomic alterations and differentiate into a plethora of subsets during retinal homeostasis and degeneration [39, 42]. Here, we showed that retinal microglia express several key purinergic receptors (P2Y_12_R, P2Y_13_R, and A_3_R) (Fig. 5b) and in addition, create an intricate and spatially arranged network in the retinal parenchyma by extending and retracting their extremely branched and motile CD39^high^/CD73^low^ processes and forming local “purinergic junctions” with CD39^low^/CD73^−^ neuronal cell bodies and CD39^high^/CD73^−^ blood vessels (Fig. 3, Movies 1 and 2). With this knowledge in mind, and knowing that extracellular ATP acts as a local chemoattractant that leads to the targeted recruitment of microglial protrusions to activated synapses [54, 55], while ATP-derived ADO plays a counteracting role in protecting retinal neurons from hyper-excitation [3, 58, 59], it may be reasonably suggested that retinal microglial cells play a pivotal role in the regulation of functional hyperemia and neurovascular coupling in the eye via coordinated control of local ATP and ADO levels.

Along with CD39, another member of this family, NTPDase2, contributes to the metabolism of ATP in the eye. This study, when analyzed together with previous data on human, rodent and zebrafish eyes, provides evidence for selective localization of NTPDase2 in the optic nerve bundles [16], Müller glia ([4, 15, 18]; Fig. 5a), as well as tubulin-βIII^+^ neuronal filaments and corneal keratocytes (Fig. 2b). Due to the high preference of NTPDase2 for the hydrolysis of ATP over ADP [51, 60], this ectoenzyme presumably has functionality in the rapid scavenging of ATP in a neuronal environment, while the subsequent degradation of ATP-derived ADP will occur with a considerable delay. We have also identified the presence of yet another enzyme, TNAP, in the mouse RGC, blood vessels and corneal epithelium (Figs. 4f and 5), as well as in the human sensory neuroretina, optic nerve head and vitreous fluid [16]. Although TNAP does not appear to contribute significantly to the metabolism of ocular adenine nucleotides, due to its surprisingly broad substrate specificity, this ectoenzyme can regulate blood clotting, bone mineralization, cartilage formation, and other cellular functions by degrading other phosphate-containing compounds, such as pyrophosphate (PP_i_) and inorganic polyphosphates [26, 30, 60]. Since TNAP has been identified among the top calcification-related genes overexpressed in the human trabecular meshwork [61], and as it is also expressed in pathological neofibrovascular tissues surgically excised from eyes with diabetic retinopathy [16], it would be interesting to evaluate the distribution of this enzyme in the eyes with pathological neovascularization and ectopic mineralization.

This work also points to the need of more careful evaluation of similarities and differences in purinergic signatures across species, which should be taken into account during studying ADO homeostasis in rodent eyes and further translating these experimental data to humans. Similar patterns of high expression of CD73 both in the human [16, 21] and rodent [22, 62] (current study) photoreceptors suggest an equally important role for this ecto-nucleotidase in governing adenosinergic signaling along the sensory retina and hence, in the development of electrical excitation in all mammalian eyes. A salient finding of this work is that pharmacological inhibition of CD73 has a fairly moderate effect on the fERG responses in dark-adapted eyes, but rendered animals became hypersensitive to continuous exposure to BL at levels that by themselves would not normally cause any adverse shifts in visual cycle or retinal structure. Taking into account data on a crucial role of ADO in regulating photoreceptor coupling [11], light and sleep signaling [10], and functional hyperemia [12] in dark-adapted eyes, it is tempting to speculate that CD73-generated ADO confers endogenous protection against light-induced phototoxicity to the retina. According to this scenario, moderate but significant decrease in b-wave waveforms in PSB-12489-treated eyes could reflect the reduced activity of photoreceptor CD73 and/or intravitreal soluble CD73 and as a consequence, insufficient activation of ARs localized on the INL and RGC facing the vitreous lumen. This impact was amplified by continuous illumination of the treated eyes, as ascertained by marked reduction of both a-wave and b-wave amplitudes (Fig. 7b). Further studies would be required to elucidate the exact mechanism(s) underlying the effects of PSB-12489 on retinal function. Taking into account the complexity of purine homeostasis in the mammalian eye (Fig. 8a), and the important role of adenosinergic signaling in retinal functioning during transition from darkness to light [11, 12], it is reasonable to conclude that inhibition of the CD73–ADO axis in dark-adapted mice could shift the balance between ocular ATP and ADO levels. Potential consequences of blocking this metabolic chain are highlighted in Fig. 8b and may particularly include the impaired hyperemic and neuroinflammatory responses mediated via activation of ARs, as well as the simultaneous accumulation of proinflammatory and cytotoxic ATP in the retinal environment.

While most of the effects of extracellular ATP and ADO are thought to be mediated via canonical signal transduction pathways, the alternative receptor-independent mechanism that is linked to the cellular uptake of ADO and its phosphorylation into ATP may play an equally important yet understudied role. Previous data demonstrated potentiation of retinal hyperemia, post-ischemic recovery, and both spontaneous and light-evoked activities of retinal neuronal cells after prevention of endogenous ADO transport and metabolism in the presence of the inhibitors of nucleoside transporters (NBTI and dipyridamole) or adenosine deaminase (EHNA) [58, 63]. Increasing the metabolic clearance of intracellular ADO in the eyes of transgenic mice overexpressing human adenosine kinase also affected circadian rhythm, manifested in the reduced slow-wave activity after sleep deprivation [10]. This intracellular purine salvage pathway might be especially relevant for RGC, because of their high ATP turnover rate and great energy demands [64]. In fact, these cells are capable of accumulating intravitreally injected [^3^H]ADO in their cellular body [43], and are particularly sensitive to the light-induced effects of ADO [44]. However, combined exposure of the mice to PSB-12489 and BL did not trigger additional apoptosis in the innermost retinal layer (Fig. S7). These observations, together with data on rather minor contribution of RGC to the mouse electroretinogram [65], allow concluding that the revealed exacerbated effects of PSB-12489 on retinal cell function were not associated with RGC death or other adverse shifts in cellular energetics in this high oxygen consumption layer.

In conclusion, these data point out the need for a more careful evaluation of the entire purinome in the mammalian eye by taking into account the complexity and redundancy of metabolic and signaling pathways involved in biological effects of ATP and ADO. Our 3D imaging workflow also provides novel insights into spatial relationships and heterotypic interactions between different cell types in the retinal environment and on this basis, suggests the important and hitherto unrecognized role of retinal microglia in the purinergic control of retinal blood flow and neuronal activity. Furthermore, data on impaired fERG responses in the mouse eyes treated with CD73 inhibitor provide evidence for a crucial role of the CD73–ADO axis in the maintenance of retinal integrity and function in “steady-state” and especially under challenging conditions induced by prolonged light illumination. As a consequence, a new enzyme-based strategy could be used to restore ADO levels and photoreceptor function in the injured retina. There is also a paucity of knowledge regarding the relationship between nucleotide-inactivating/ADO-producing and counteracting ATP-regenerating ectoenzymes, as well as intracellular purine salvage pathways in the eye. Improving our knowledge in this field may be useful for understanding the role of purine homeostasis in ocular (dys)functions and on this basis, developing effective new strategies for the treatment of retinal degeneration and other vitreoretinal diseases.

## Supplementary Information

Below is the link to the electronic supplementary material.

Supplementary file1 (DOCX 5687 KB)

Supplementary file2 (MP4 5120 KB)

Supplementary file3 (MP4 4925 KB)

## Data Availability

The datasets generated during the current study are available from the corresponding author on reasonable request.
