# Supplementary material for: CD73 controls ocular adenosine levels and protects retina from light-induced phototoxicity
Source: Cell Mol Life Sci. 2022 Feb 25;79(3):152. doi: 10.1007/s00018-022-04187-4 (PMC8881442; doi:10.1007/s00018-022-04187-4)
Supplement: Supplementary file 1 — Supplementary file1 (DOCX 5687 KB) [file 18_2022_4187_MOESM1_ESM.docx]

**Supplementary data for the manuscript: “CD73 controls ocular adenosine levels and protects retina from light-induced phototoxicity”**

Karolina Losenkova^1^, Akira Takeda^1^, Symantas Ragauskas^2^, Marc Cerrada-Gimenez^3^, Maria Vähätupa^3^, Simon Kaja^3,4^, Marius L. Paul ^1,5^, Constanze C. Schmies^5^, Georg Rolshoven^5^, Christa E. Müller^5^, Jouko Sandholm^6^, Sirpa Jalkanen^1^ , Giedrius Kalesnykas^2,3^ and Gennady G. Yegutkin^1🖂^

^1^ MediCity Research Laboratory and InFLAMES Flagship, University of Turku, Turku, Finland;

^2^ Experimentica UAB, Vilnius, Lithuania;

^3^ Experimentica Ltd., Kuopio, Finland;

^4^ Department of Ophthalmology, Loyola University Chicago, Stritch School of Medicine, Maywood, IL, USA

^5^ Pharma Center Bonn, Pharmaceutical Institute, Pharmaceutical and Medicinal Chemistry, University of Bonn, Bonn, Germany

^6^ Turku Bioscience Centre, University of Turku and Åbo Akademi University, Turku, Finland

**Online supplemental material**

*Table S1* provides detailed information on primary antibodies used in this study. *Table S2* summarizes fERG stimulus parameters. *Table S3* shows the script for quantitative analysis of photoreceptor AMPase activity. *Fig. S1* is associated with *Fig. 2* and provides supporting information about the distribution of CD39 in the mouse retinal vessels, choriocapillaris and cornea. *Fig. S2* summarizes competitive TLC data showing the ability of CD73 inhibitors to inhibit the hydrolysis of [^3^H]AMP by human and mouse sera. *Fig. S3* is associated with *Fig. 7* and provides additional information about a-wave and b-wave amplitudes before and after exposure of dark-adapted mice to bright light. *Fig. S4* shows quantitative analysis of histological data on AMPase photoreceptor activity in the eyes of mice exposed to CD73 inhibitor and BL. *Fig. S5* shows immunofluorescence analysis of the distribution of CD73 in the mouse eyes exposed to CD73 inhibitor and BL. *Fig. S6* shows histological analysis of the integrity of retinal layers in the mouse eyes exposed to CD73 inhibitor and BL. *Fig. S7* shows that impaired functional responses of retinal cells in PSB-12489-treated mice were not accompanied by death and apoptosis of retinal ganglion cells. *Movie 1* is associated with *Fig. 3A* and allows better visualization of direct interaction between microglial cell processes and blood vessel wall in the mouse retina. *Movie 2* is associated with *Fig. 3B* and allows stereoscopic visualization of microglia-neuron purinergic junction in the mouse retina.

**Files in the Data Supplement:**

**Table S1.** Primary antibodies used for immunofluorescence staining and flow cytometry analysis

**Table S2.** fERG stimulus parameters

**Table S3.** QuPath v.0.2.3 script for the analysis of photoreceptor AMPase activity

**Fig. S1** Immunofluorescence analysis of the distribution of CD39 in the mouse retina and cornea.

**Fig. S2** Competitive analysis of [^3^H]AMP hydrolysis by soluble CD73 in the human and mouse sera.

**Fig. S3.** Electrophysiological analysis of retinal activity recorded before and after exposure of BALB/c mice to CD73 inhibitor and bright light.

**Fig. S4.** Enzymatic *in situ* analysis of photoreceptor AMPase activity in the mouse eyes exposed to CD73 inhibitor and bright light.

**Fig. S5.** Immunofluorescence analysis of the distribution of CD73 in the mouse eyes exposed to CD73 inhibitor and bright light.

**Fig. S6.** Histological analysis of the integrity of retinal layers in the mouse eyes exposed to CD73 inhibitor and bright light.

**Fig. S7.** Inhibition of CD73 activity in dark-adapted eyes was not accompanied by changes in apoptosis in the ganglion cell layer.

**Movie 1.** Visualization of direct interaction between microglial cell processes and blood vessel in the mouse retina.

**Movie 2.** Visualization of microglia-neuron somatic junctions in the mouse retina by using multiplexed 3D imaging approach.

**Table S1.** Primary antibodies used for immunofluorescence stainings and flow cytometry analysis

| **Target** | **Clone and catalogue number** | **Host** | **Dilution** | **Manufacturer** |
| --- | --- | --- | --- | --- |
| CD73 | Anti-rat CD73 Ab (rNu9_L_-I5) | Rabbit | 1:300 | Ectonucleotidases^1^ |
| CD73 (Neg. Co.) | Pre-immune serum (rNu9_L_-PI) | Rabbit | 1:300 | Ectonucleotidases^1^ |
| CD39 | Anti-mouse CD39 Ab (mN1-2_C_I5) | Guinea pig | 1:600 | Ectonucleotidases^1^ |
| CD39 (Neg. Co.) | Pre-immune serum (mN1-2_C_PI) | Guinea pig | 1:600 | Ectonucleotidases^1^ |
| NTPDase2 | Anti-mouse NTPDase2 Ab (mN2-36_L_I6) | Rabbit | 1:300 | Ectonucleotidases^1^ |
| NeuN | Anti-NeuN mAb (A60/MAB377) | Mouse | 1:300 | Merck Millipore® |
| Rhodopsin | Anti-Rhodopsin mAb (RET-P1/MAB5316) | Mouse | 1:800 | Merck Millipore® |
| Vimentin | Anti-vimentin Ab (919101) | Chicken | 1:800 | Biolegend Inc |
| CD31 | Alexa Fluor^®^ 488 (102414) or APC (102510) conjugated anti-CD31 mAb | Rat | 1:100 | Biolegend Inc |
| P2Y_12_R | Anti-mouse P2Y_12_R Ab (55043A) | Rabbit | 1:300 | AnaSpec Inc |
| P2Y_12_R | PE-conjugated anti-mouse P2Y_12_R Ab (848003) | Rat | 1:100 | BD Biosciences |
| NG2 | Anti-mouse NG2 Ab (AB5320) | Rabbit | 1:300 | Merck Millipore® |
| Tubulin-βIII | NL493-conjugated anti-tubulin-βIII mAb (TuJ-1/NL1195G) | Mouse | 1:100 | R&D Systems |
| SMA-α | Cy3™-conjugated anti-SMA-α mAb (1A4) | Mouse | 1:100 | Sigma-Aldrich |
| CD45 | APC-Cy7 (557659)- or BV450 (560501)-conjugated anti-CD45 Ab | Rat | 1:100 | BD Biosciences |
| CD11b | APC-Cy7-conjugated anti-CD11b Ab (557657) | Rat | 1:100 | BD Biosciences |

^1^ <http://ectonucleotidases-ab.com/>

Abbreviations: *APC* Allophycocyanin, *BV450* Brilliant Violet 421™, *Cy* Cyanine dye, *GFAP* glial fibrillary acidic protein, *Iba1* ionized calcium binding adaptor molecule-1, *Neg. Co*. negative control, *NeuN* neuronal nuclear protein, *NL493* Northern Lights™ 493, *PE* Phycoerythrin, *PI* pre-immune serum, *SMA-α* smooth muscle actin-α

**Table S2.** fERG stimulus parameters

| **Flash intensity, cd*s/m^2^** | **Interstimulus interval, s** | **Repeats** |
| --- | --- | --- |
| 0.001 | 3 | 6 |
| 0.005 | 3 | 6 |
| 0.01 | 3 | 6 |
| 0.1 | 5 | 6 |
| 1 | 10 | 6 |
| 3 | 15 | 6 |
| 10 | 15 | 6 |

**Table S3.** QuPath v.0.2.3 script for the analysis of photoreceptor AMPase activity

| setColorDeconvolutionStains('{"Name" : "H-DAB default", "Stain 1" : "Hematoxylin", "Values 1" : "0.65111 0.70119 0.29049 ", "Stain 2" : "DAB", "Values 2" : "0.26917 0.56824 0.77759 ", "Background" : " 255 255 255 "}'); |
| --- |
| selectDetections(); |
| runPlugin('qupath.lib.algorithms.IntensityFeaturesPlugin', '{"pixelSizeMicrons": 2.0, "region": "ROI", "tileSizeMicrons": 25.0, "colorOD": false, "colorStain1": false, "colorStain2": true, "colorStain3": false, "colorRed": false, "colorGreen": false, "colorBlue": false, "colorHue": false, "colorSaturation": false, "colorBrightness": false, "doMean": true, "doStdDev": true, "doMinMax": true, "doMedian": true, "doHaralick": false, "haralickDistance": 1, "haralickBins": 32}'); |

**
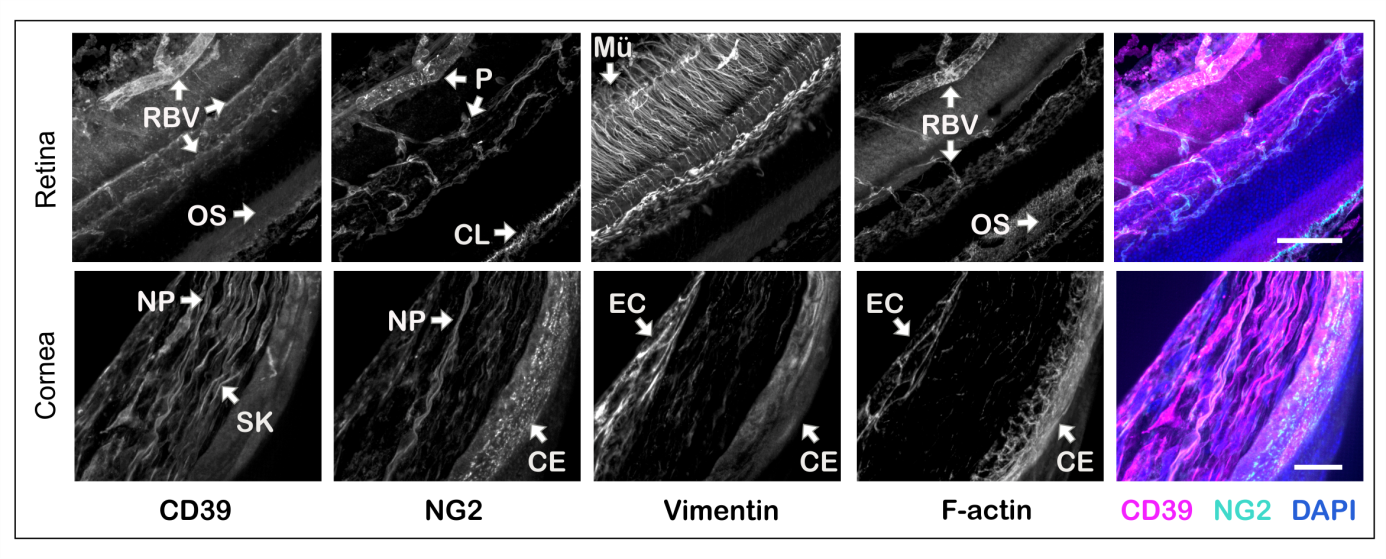
**

**Fig. S1** Immunofluorescence analysis of the distribution of CD39 in the mouse retina and cornea.

LMA-embedded sections of the mouse eye were stained in free-floating assays with anti-CD39 antibody and molecular markers of pericytes (NG2), the intermediate filaments of Müller cells (vimentin), and filamentous actin (F-actin, Alexa Fluor 546 Phalloidin), as indicated. The 3D images of the medial region of retina and cornea were captured using a 3i spinning disk confocal microscope. Maximum intensity projections for individual channel are shown in grayscale. The right panel displays merged images of selected channels with nuclei counterstained with DAPI. *CE* corneal epithelium, *CL* choroidal layer, *EC* endothelial cells, *Mü* Müller cells, *NP* neuronal processes, *OS* outer segments of photoreceptors, *P* pericytes, *RBV* retinal blood vessels, *SK* stromal keratocytes. Scale bars: 50 µm (upper panels), and 20 µm (lower panels).

**
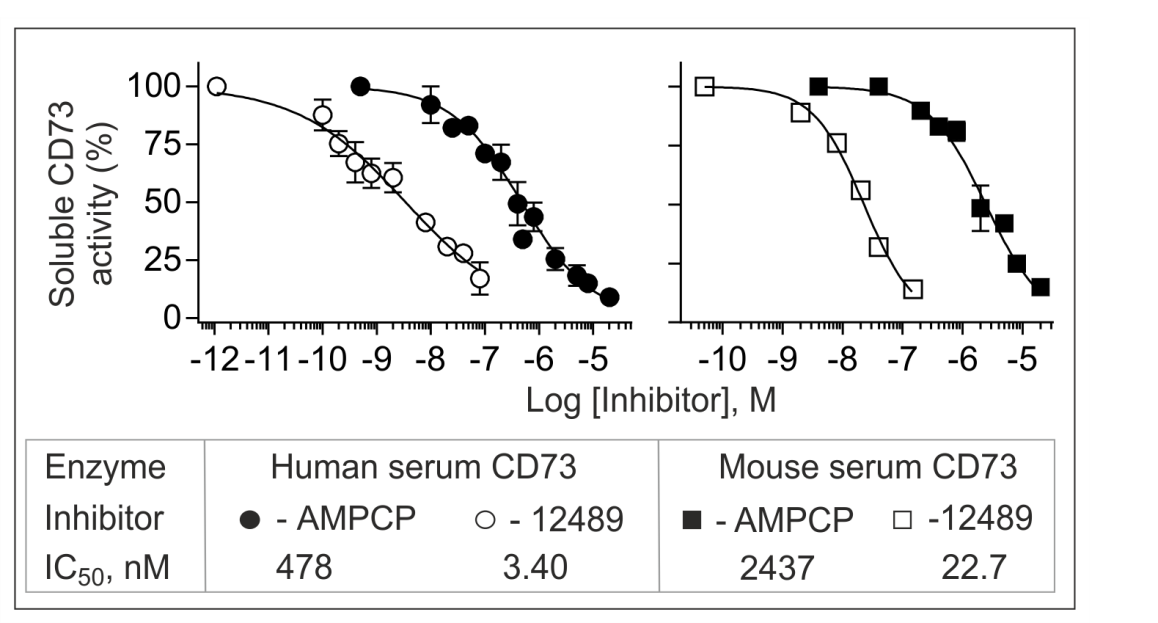
**

**Fig. S2** Competitive analysis of [^3^H]AMP hydrolysis by soluble CD73 in the human and mouse sera. Soluble CD73 activity was assayed by TLC by incubating human and mouse serum samples with 40 μM [^3^H]AMP and increasing concentrations of AMPCP and PSB-12489 (*12489*). The results are plotted as the percentage of maximal [^3^H]AMP hydrolysis determined in the absence of inhibitors (mean ± SEM; n=3-6). IC_50_ values were calculated from one-site competition curves constructed using nonlinear least-squares curve fitting and shown as nmol/L.

**
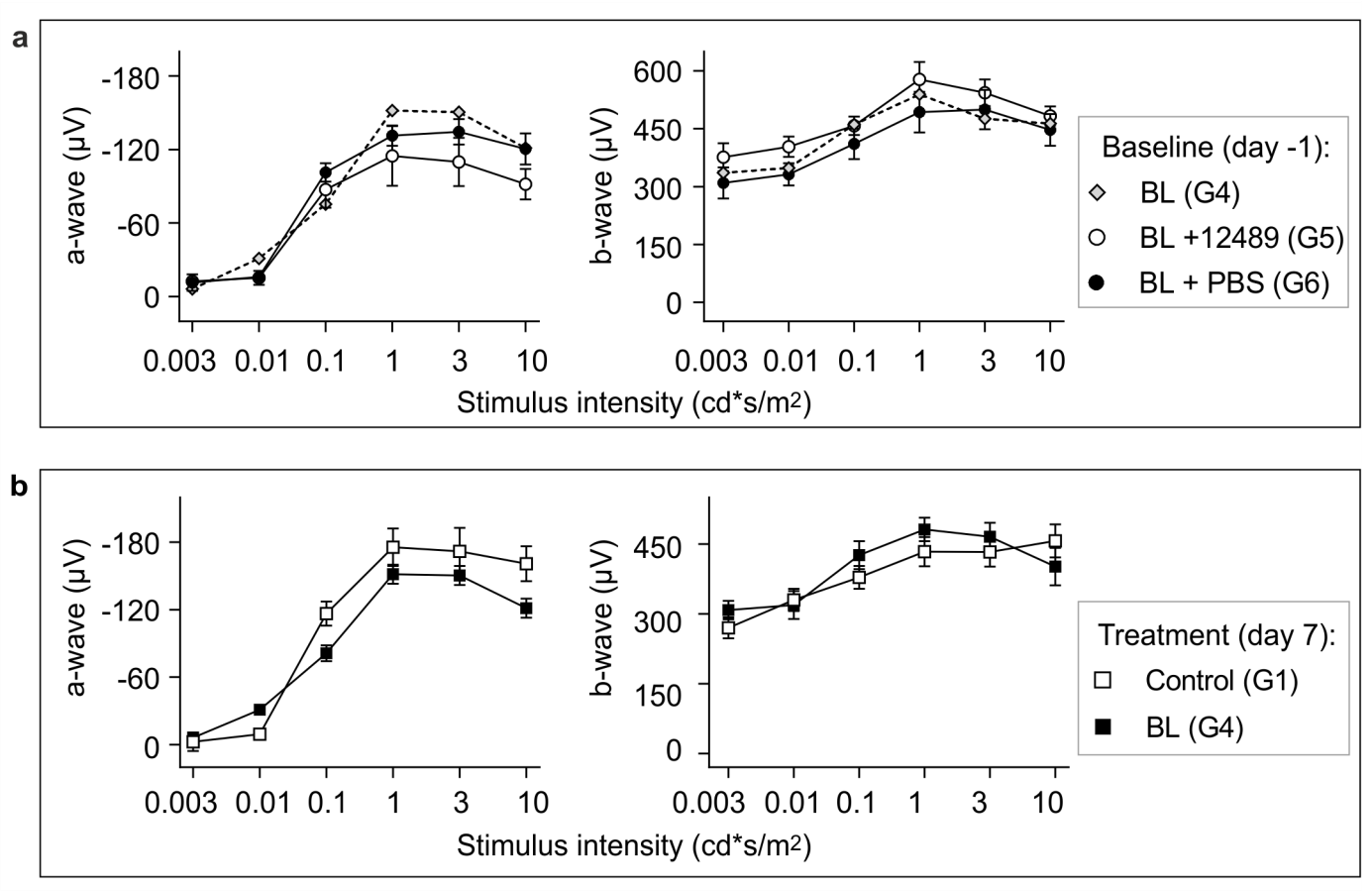
**

**Fig. S3** Electrophysiological analysis of retinal activity recorded before and after exposure of BALB/c mice to CD73 inhibitor and bright light. Male BALB/c mice were divided into groups, which either remained untreated or received a single intravitreal injection of PSB-12489 (*12489*) or vehicle (PBS). The animals were kept in transparent plastic cages without any treatment (Control) or additionally exposed to continuous bright light (BL), as outlined in Figure 7a. The a-wave and b-wave amplitudes were recorded both at baseline (**a**) and seven days after the treatment (**b**), as indicated (mean ± SEM; n=6).


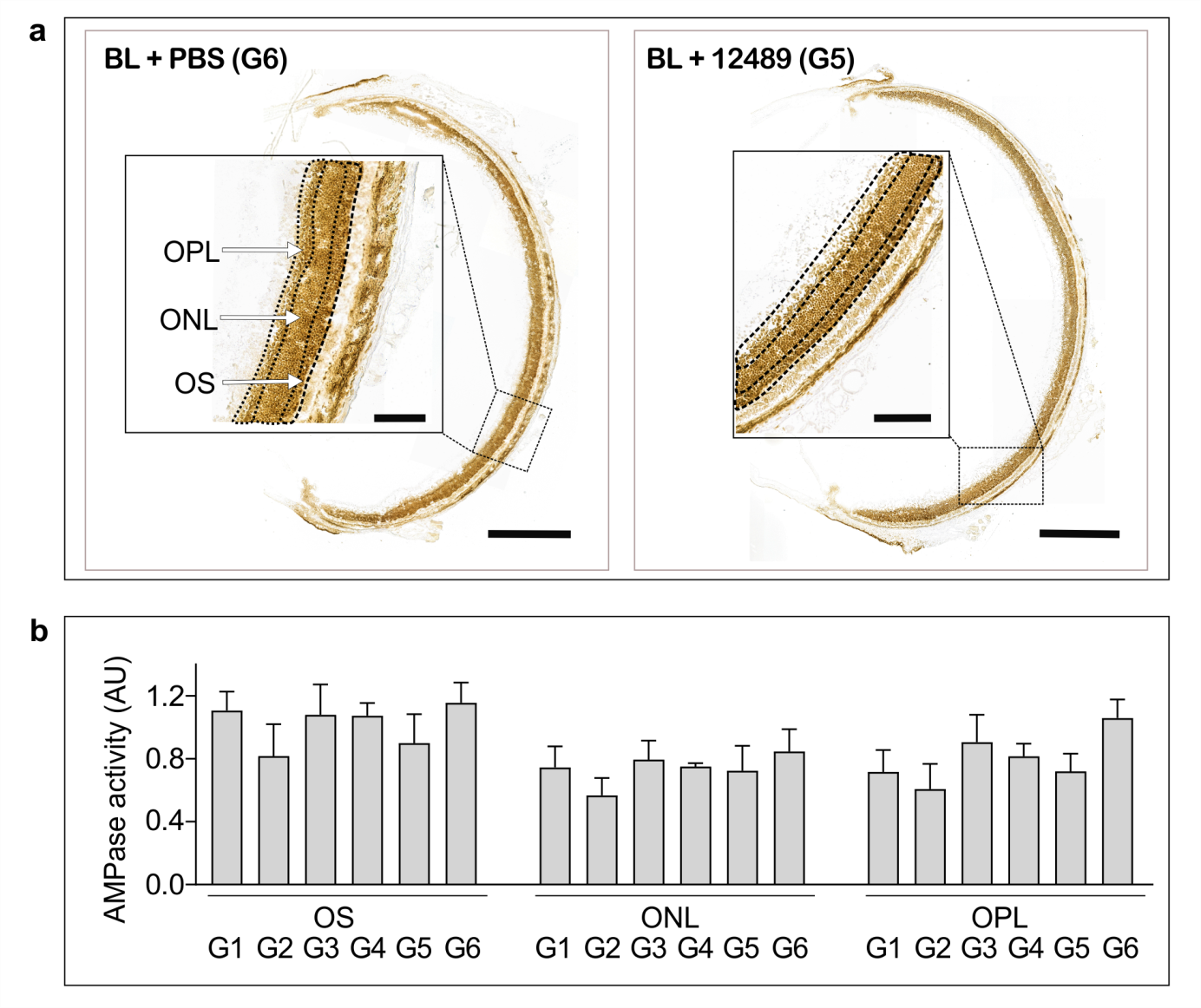


**Fig. S4** Enzymatic *in situ* analysis of photoreceptor AMPase activity in the mouse eyes exposed to CD73 inhibitor and bright light. BALB/c mice remained untreated or received a single intravitreal injection of PBS-12489 or PBS, and were subsequently exposed to BL, as outlined in Figure 7a. Seven days after the treatment, the eyeballs were enucleated and processed for histological analysis. (**a**) CD73 activity was assayed by incubating eye cryosections with AMP in the presence of Pb(NO_3_)_2_, followed by microscopic detection of the nucleotide-derived inorganic phosphate as a brown precipitate. Representative images of AMP staining in the eyes treated with PBS (left) and PSB-12489 (right), and the medial areas of the retinas selected for quantitative analysis of photoreceptor AMPase (insets) are shown. Scale bars, 500 µm and 100 µm (inset). (**b**) Photoreceptor CD73 activity was determined by measuring AMP-specific intensities in the OS of photoreceptors, ONL, and OPL. The results are expressed as mean pixel intensities per selected area. *ONL* outer nuclear layer, *OPL* outer plexiform layer, *OS* outer segments.


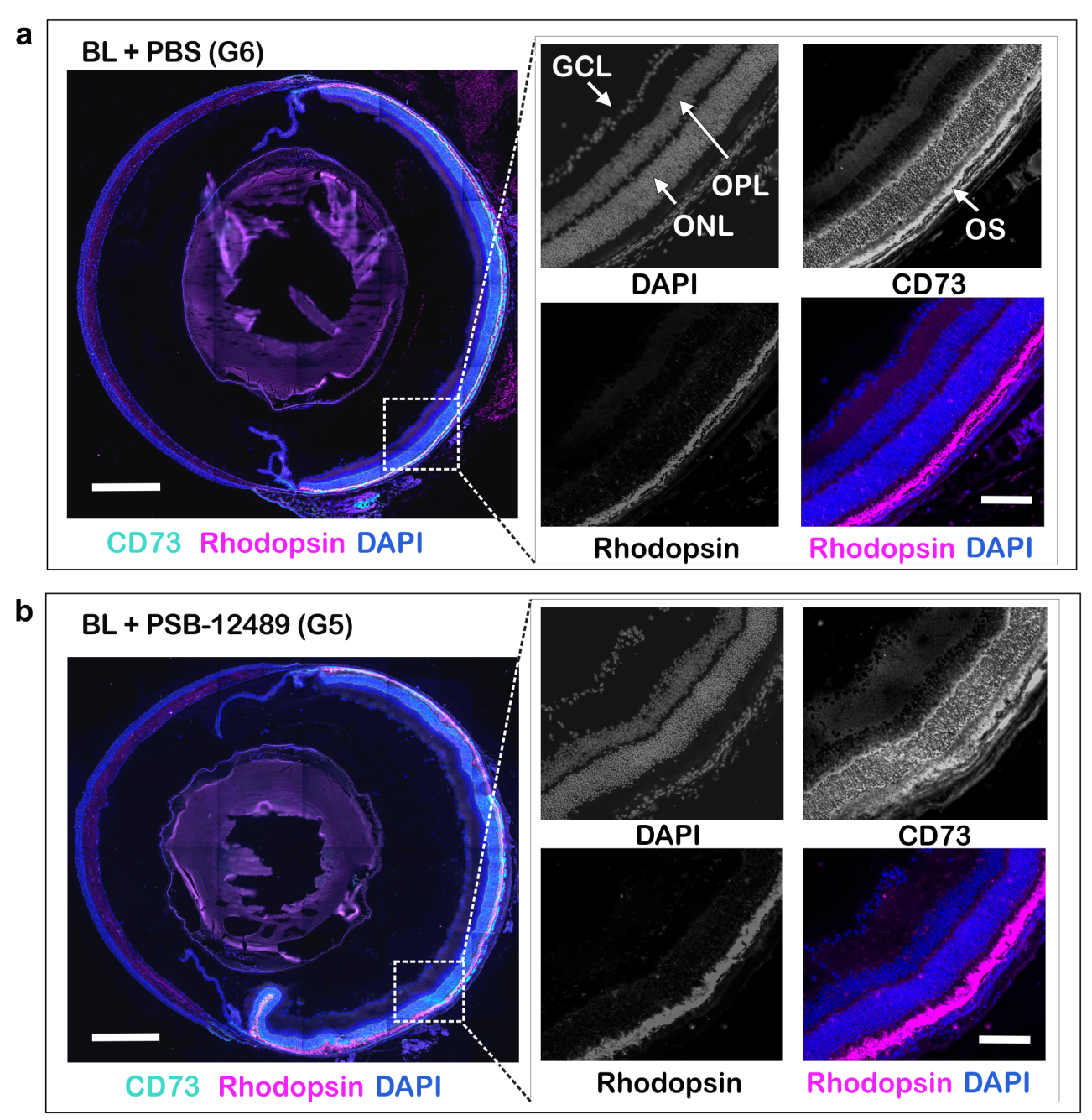


**Fig. S5** Immunofluorescence analysis of the distribution of CD73 in the mouse eyes exposed to CD73 inhibitor and bright light. BALB/c mice received a single intravitreal injection of PBS (**a**) or CD73 inhibitor PSB-12489 (**b**), and subsequently exposed to BL, as outlined in Figure 7a. Seven days after the treatment, the eyeballs were enucleated and processed for immunofluorescence analysis of CD73 expression in photoreceptor cell layer. Tissue-specific distribution of CD73, marker of rod photoreceptors rhodopsin and cell nuclei (DAPI) are shown in the insets in grayscale, with the lower right panels displaying merged colorful images for selected channels. *GCL* ganglion cell layer, *ONL* outer nuclear layer, *OPL* outer plexiform layer, *OS* outer segments. Scale bars: 500 µm (a,b) and 100 μm (right-hand insets in panels a and b).

**
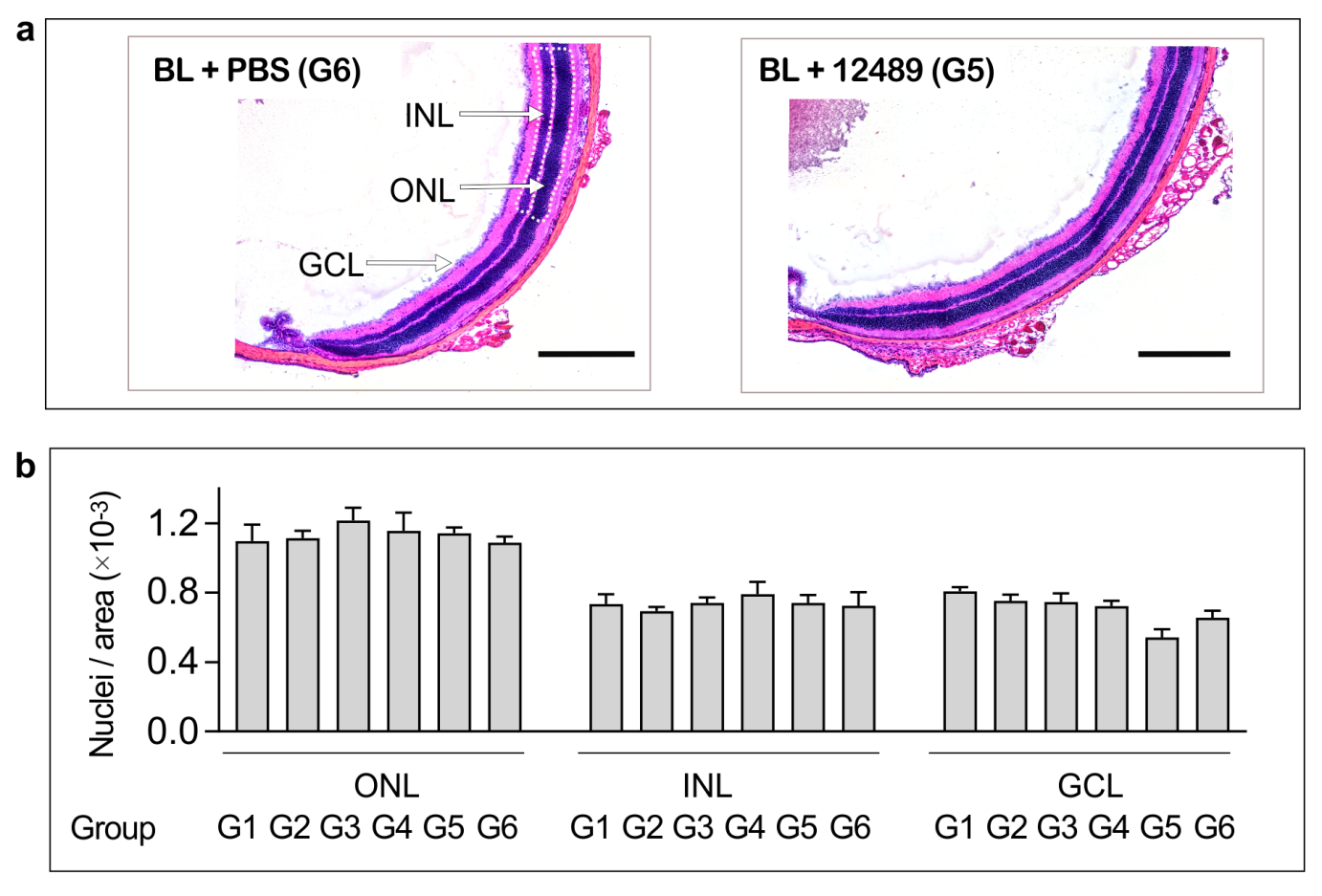
**

**Fig. S6** Histological analysis of the integrity of retinal layers in the mouse eyes exposed to CD73 inhibitor and bright light. BALB/c mice remained untreated or received a single intravitreal injection of CD73 inhibitor PSB-12489 (*12489)* or vehicle (PBS), and were subsequently exposed to BL, as outlined in Figure 7a. Seven days after the treatment, the eyeballs were enucleated and processed for histological analysis. (**a**) Eye cryosections were stained with hematoxylin and eosin. Representative images of retinal staining in the eyes treated with PBS (left) and PSB-12489 (right) are shown. Scale bars, 500 µm. (**b**) The integrities of the retinal layers were interrogated by quantitative analyses of cell nuclei in the ONL, INL, and also in GCL and expressed as nuclei counts per area of the layer. Data are presented as mean ± SEM (n=3). *GCL* ganglion cell layer, *INL* inner nuclear layer, *ONL* outer nuclear layer.

**
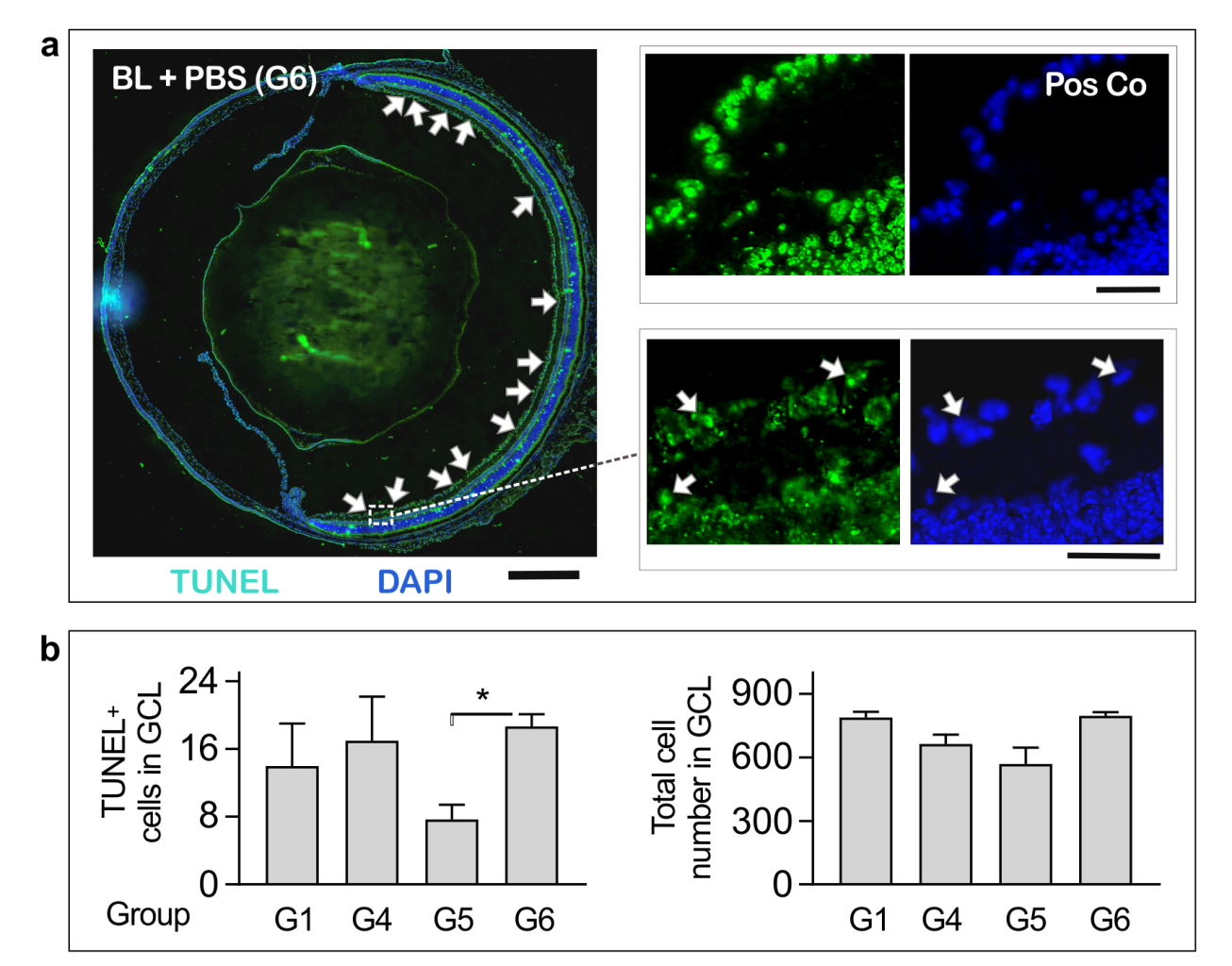
**

**Fig. S7** Inhibition of CD73 activity in dark-adapted eyes was not accompanied by changes in apoptosis in the ganglion cell layer. BALB/c mice remained untreated or received a single intravitreal injection of CD73 inhibitor PSB-12489 (*12489)* or vehicle (PBS), and were subsequently exposed to BL, as outlined in Figure 7a. (**a**) The effect of CD73 on retinal cell apoptosis was evaluated by using TUNEL Apoptosis Assay, as described in the Materials and Methods. The arrows point out TUNEL-positive apoptotic cells in the innermost GCL. The upper right inset displays representative retinal area in the positive control sample (*Pos Co*), with was pre-treated with DNase-I in order to induce DNA strand breaks. (**b**) Total ganglion cell numbers and numbers of TUNEL-positive apoptotic cells in GCL of the treated eyes were quantified using QuPath v.0.3.0 software and Stardist deep learning platform via Fiji-ImageJ, respectively. The results are presented as mean ± SEM (n=3). *P<0.05, determined by unpaired two-tailed Student’s t-test. Scale bars: 500 µm (a), and 40 µm (a, insets).

**Movie 1.** Visualization of direct interaction between microglial cell processes and blood vessel in the mouse retina.

LMA-embedded sections of the mouse eye were co-stained in free-floating assays with anti-CD39 antibody (magenta) and molecular markers of blood endothelial cells (CD31, green) and microglial cells (P2Y_12_R, cyan). Serial confocal images along the z-axis were captured using a 3i CSU-W1 spinning disk confocal microscope with Plan-Apochromat 20×/0.8 objective (Intelligent Imaging Innovations GmbH). 3D image was subsequently rendered into a movie using Imaris 8.4 software (Bitplane), and exported to mp4 format. For single channel view of the captured 3D reconstructed images, see Fig. 3a.

**Movie 2.** Visualization of microglia-neuron somatic junctions in the mouse retina by using multiplexed 3D imaging approach.

LMA-embedded sections of the mouse eye were co-stained in free-floating assays with anti-CD39 antibody (magenta) and molecular markers of neuronal cell bodies (NeuN, green) and microglial cells (P2Y_12_R, grey). Serial confocal images were captured using a 3i CSU W1 spinning disk confocal microscope with Plan-Neofluar 63×/1.4 oil objective (Intelligent Imaging Innovations GmbH). 3D image stack was subsequently rendered into a movie using Imaris 8.4 software (Bitplane). For single channel view of the captured 3D reconstructed images, see Fig. 3b.
